# The Alignment Paradox of Medical Large Language Models in Infertility Care: Decoupling Algorithmic Improvement From Clinical Decision-Making Quality

**DOI:** 10.2196/97221

**Published:** 2026-09-21

**Authors:** Dou Liu, Ying Long, Sophia Zuoqiu, Di Liu, Kang Li, Kaipeng Xie, Runze Yang, Yiting Lin, Hanyi Liu, Rong Yin, Tian Tang

**Affiliations:** 1 Department of Obstetrics and Gynecology, West China Second University Hospital of Sichuan University, Chengdu, Sichuan, China; 2 Department of Industrial and Operations Engineering, University of Michigan, Ann Arbor, MI, United States; 3 Key Laboratory of Birth Defects and Related Diseases of Women and Children, Sichuan University, Chengdu, Sichuan, China; 4 Reproductive Medical Center, Department of Obstetrics and Gynecology, West China Second University Hospital of Sichuan University, Chengdu, Sichuan, China; 5 Department of Industrial Engineering, Sichuan University, No.37, Guoxue Lane, Wuhou District, Chengdu, Sichuan, China, 86 17208267909; 6 West China Biomedical Big Data Center, West China Hospital, Sichuan University, Chengdu, Sichuan, China; 7 Med-X Center for Informatics, Sichuan University, Chengdu, Sichuan, China; 8 West China School of Medicine, Sichuan University, Chengdu, Sichuan, China

**Keywords:** large language models, clinical alignment, assisted reproductive technology, reproductive medicine, infertility treatment, reinforcement learning from human feedback, clinical reasoning, medical artificial intelligence

## Abstract

**Background:**

Large language models (LLMs) have been proposed as decision-support tools in assisted reproductive technology (ART), but it remains unclear whether posttraining alignment strategies translate into clinically acceptable decision support. Outcome-based benchmarks may reward token-level correctness while overlooking the reasoning quality that clinicians rely on.

**Objective:**

This study aimed to evaluate whether 4 mainstream alignment paradigms for medical LLMs (supervised fine-tuning [SFT], direct preference optimization [DPO], group relative policy optimization [GRPO], and in-context learning [ICL]) produce comparable algorithmic and clinical alignment (SFT vs GRPO) when used for infertility diagnosis and treatment planning.

**Methods:**

This retrospective single-center study used 8201 deidentified electronic health records from West China Second University Hospital, collected between January 2020 and December 2022 (mean age 31.79, SD 4.63 y). All 4 strategies were built on a shared open-source biomedical backbone. Evaluation comprised the following 2 tiers: (1) automatic field-level metrics (accuracy, macro-*F*_1_, and mean absolute error [MAE]) on 5 structured decision fields (infertility type, initial diagnosis, ART strategy, controlled ovarian stimulation [COS] regimen, and gonadotropin starting dose); and (2) blinded independent expert review by 2 reproductive medicine specialists on 100 paired cases across 4 clinical dimensions (reasoning capability, diagnostic accuracy, treatment feasibility, and hallucination). Automatic field-level evaluation included all 4 strategies, whereas blinded expert review was restricted to the SFT versus GRPO contrast.

**Results:**

GRPO achieved the highest average automatic performance (eg, infertility type accuracy=92.57%, COS regimen accuracy=62.36%, ART strategy accuracy=76.49%, and gonadotropin dose MAE=44.94). However, in blinded expert review, the conservative SFT baseline showed directionally higher expert ratings than GRPO on reasoning capability and treatment feasibility; diagnostic-accuracy differences were not significant. In the 3-way best-response comparison including the original physician-charted plan, the SFT baseline was selected as the best response in 51.2% (102.3/200) of cases compared with 26.2% (52.4/200) for GRPO, and 22.6% (45.3/200) for the charted plan. This result reflects preference within the standardized review format, not evidence that model-generated decisions are clinically superior to physician decision-making. Hallucination rates were 15% (GRPO) and 18.5% (SFT), indicating that higher automatic performance did not eliminate clinically unsupported content and that neither model is ready for clinical deployment. Subgroup analyses showed GRPO improved *F*_1_ in in vitro fertilization (IVF) and preimplantation genetic testing (PGT) but decreased *F*_1_ in intracytoplasmic sperm injection (ICSI) cases, where male-factor information was largely captured only in unstructured fields.

**Conclusions:**

Outcome-based metrics alone are insufficient proxies for clinical utility in ART decision support. Algorithmic improvement and clinical alignment suggest a possible decoupling; a phenomenon we term the alignment paradox. However, because the clinical review was based on 2 reproductive medicine specialists with marginal interrater agreement, the clinical-alignment findings should be interpreted as exploratory. External multicenter validation is required before clinical deployment.

## Introduction

Infertility is one global health issue that is experienced by 1 in 6 people at a certain stage in their lives, according to the World Health Organization (WHO) [1]. Assisted reproductive technology (ART) is a common clinical pathway for these cases if conventional treatments, such as cycle regulation or ovulation induction, do not work [2]. ART needs to consider the complexity of high-dimensional data to make an effective treatment plan. Clinicians must also determine the controlled ovarian stimulation (COS) protocol and the gonadotropin starting dose, which are generally sensitive to the patient’s physiological state. Consequently, ART decision-making is a multistage, high-dimensional, and evidence-driven process that is both time-consuming and cognitively demanding. Clinical outcomes are highly dependent on accurate reasoning, where inappropriate treatment selection may have negative outcomes such as reduced cycle success rates, increased financial and emotional burden, and even increased the risk of severe complications such as ovarian hyperstimulation syndrome (OHSS) [3]. These decisions rely heavily on individual clinical experience, leading to substantial interphysician and/or intercenter variability. The possibility of training generalization for novice physicians is thus more challenging. Moreover, ART decision-making faces structural challenges due to imbalanced medical resources. For top-tier medical centers that have large patient volumes, inducing severe decision fatigue and potential safety risks among experienced specialists [4]. On the other hand, primary care often lacks the specialized reasoning capability required for such high-dimensional decision-making, leading to significant intercenter variability in care quality. This “capability-capacity gap” calls for the need for expert-level clinical decision support systems. The inherently complex nature of ART makes it both a representative real-world medical decision environment and a sensitive setting. Thus, failures of model alignment are likely to be amplified and clinically consequential, if present.

Large language models (LLMs) have rapidly advanced across domains, while limited studies address the issues about how their advanced alignment interacts with the hierarchical, high-stakes reasoning in real-world medicine, especially reproductive medicine. Currently, typical alignment methods usually involve various reinforcement learning variants such as reinforcement learning from human feedback (RLHF), group relative policy optimization (GRPO), and direct preference optimization (DPO), which have shown remarkable improvement within some general areas by using verifiable rewards or preference signals to optimize the policy model [5-9]. For example, after adopting the GRPO, the performance of DeepSeek-R1 on math questions significantly outperformed the baseline [8]. However, clinical decision-making presents fundamentally different challenges; the reasoning chains are often long and multidimensional, where signals are noisy and unverifiable, and clinicians commonly require explanation quality and the entire plan’s feasibility rather than solely focusing on the single field output correctness alone. These properties suggest a structural tension between *algorithmic alignment* and *clinical alignment*, which has not been systematically examined.

The domain-specific medical LLMs, such as Med-PaLM (Google), MedGemma (Google), and Lingshu (Alibaba), were proven to exhibit profound potential in clinical problems, ranging from text classification to clinical image reports [10-16]. Some models demonstrate capabilities with high performance in the answer correctness on benchmark datasets. Despite their outstanding performance, when these state-of-the-art (SOTA) models are applied to specialized real-world clinical cases to support the realistic diagnosis operation, they seem to encounter some obstacles [17,18]. Although SOTA models' generated responses were preferred to those provided by generalist physicians, they still did not outperform domain-specific specialists, indicating limited utility in addressing highly specialized routine clinical problems [19]. Moreover, the untransparent reasoning processes limit their clinical adoption, despite evidence that explainable AI enhances trust and interpretability [20-23]. While supervised fine-tuning (SFT) remains the dominant paradigm in medical model development [17,24], limited data and incomplete supervision motivate increasing reliance on posttraining alignment [12,25,26]. Although RLHF-style optimization is increasingly used, little is known about whether improvements in algorithmic metrics translate into enhanced interpretability, trust, or multistep clinical reasoning. Additionally, few studies have evaluated how different alignment paradigms behave in real-world clinical decision environments. Reasoning quality in these scenarios directly affects patient safety. These alignment strategies mainly optimize for algorithmic rewards and often fail to capture the interpretability and process-oriented reasoning distinct to medical practice. Specifically, standard alignment targets may overlook the feasibility of the treatment plan, resulting in model failures in obtaining the trust necessary for clinical application. A fundamental research gap is whether stronger posttraining alignment produces more clinically aligned models.

In this study, we propose the following 2 key research questions (RQs):

- RQ1: How do SFT and reinforcement-based alignment strategies (eg, GRPO and DPO) suffice to capture the high-dimensional complexity of real-world ART treatment and enhance decision-making?
- RQ2 (the paradox): Is there a possible decoupling between “algorithmic alignment” (ie, reward maximization) and “clinical alignment” (ie, human trust) in high-risk areas (ie, reproductive medicine)?

**Figure 1.**
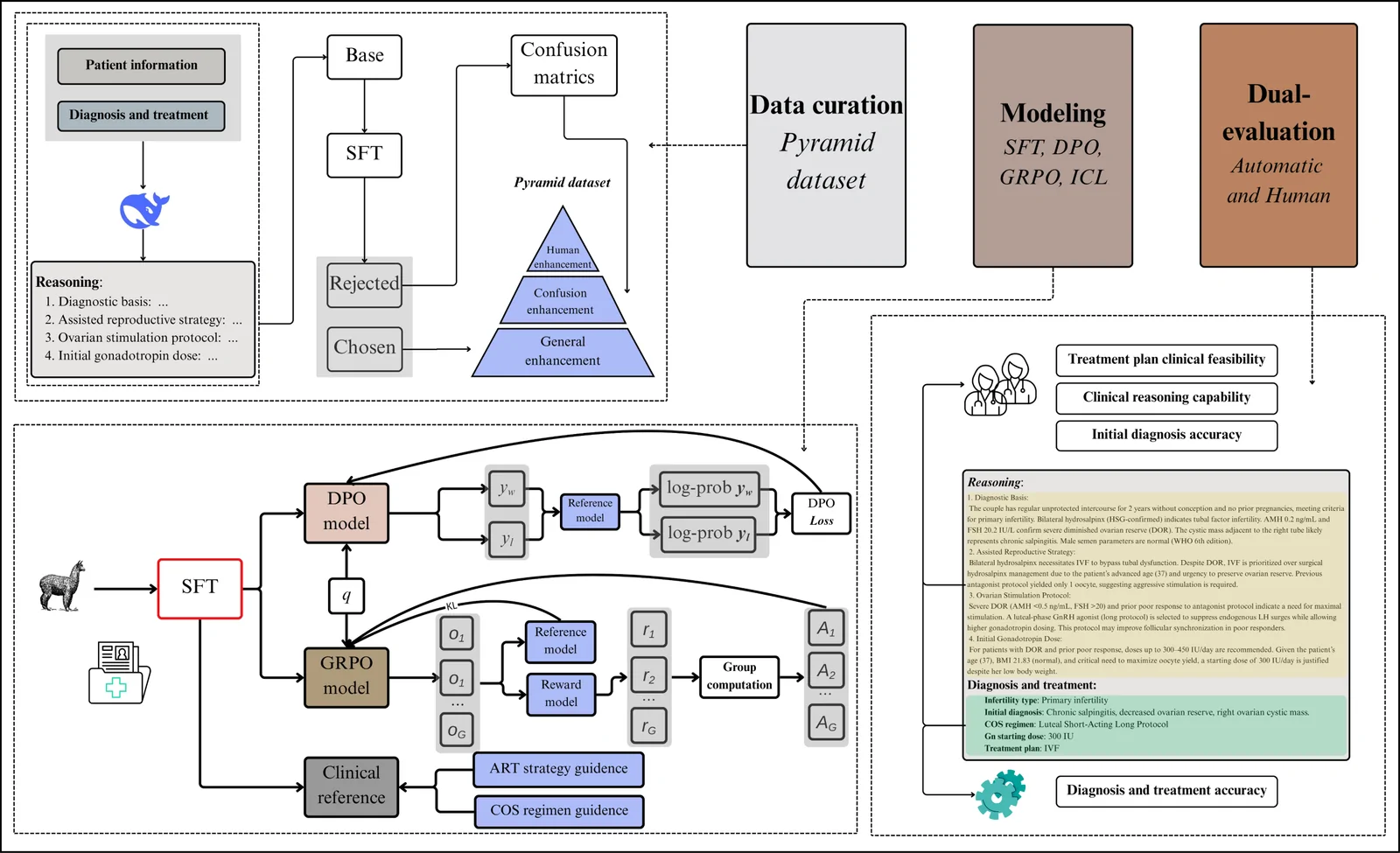
Study workflow for evaluating four alignment paradigms for large language models in assisted reproductive technology decision support. A single-center retrospective dataset of 8201 deidentified electronic health records from the reproductive medical center, West China Second University Hospital, Sichuan University (Chengdu, China; January 2020-December 2022) was used to train, validate, and test supervised fine-tuning (SFT), direct preference optimization (DPO), group relative policy optimization (GRPO), and in-context learning (ICL) variants of a shared biomedical backbone. A pyramid-curated alignment dataset combining general, model-confused, and expert-refined cases was used for post-training. Outputs were evaluated by a dual framework combining automatic field-level metrics and blinded independent expert review by reproductive medicine specialists.

We define the “alignment paradox” as follows: in clinical decision-support settings, posttraining optimization that demonstrably improves algorithmic performance on structured outcome metrics (ie, algorithmic alignment) may simultaneously degrade physician-perceived reasoning quality and treatment feasibility (ie, clinical alignment). In other words, a model may become more accurate on paper while becoming less trustworthy in practice. This paradox is clinically significant because, in high-stakes fields, such as reproductive medicine, clinicians rely not only on the correctness of the final recommendation but also on the transparency and coherence of the reasoning process that leads to it. If optimization erodes this process, it undermines the very foundation of clinical trust, regardless of outcome-level gains. To address these questions, we systematically compare 4 representative alignment paradigms: SFT, DPO, GRPO, and in-context learning (ICL), using a large-scale real-world dataset of more than 8000 infertility cases. To enhance robustness on challenging and long-tail cases, we construct a hierarchical “pyramid” dataset that emphasizes diverse difficulty levels and decision structures. Most importantly, we introduce a dual-evaluation framework that integrates automated metric-based assessment with blinded independent doctor-in-the-loop evaluations, in which the physicians, the model identities, and the case-model assignments are all blinded, enabling us to directly examine the relationship between algorithmic optimization and clinician-centered preferences. This framework allows us to compare both alignment strategies’ performance and the relationship between algorithmic and clinical alignment, which is the core of the alignment paradox that we investigate in this study. The overview of the alignment and evaluation framework is presented in Figure 1.

## Methods

### Data Source

This study includes 19,800 electronic health records (EHRs) data from West China Second University Hospital, spanning from January 2020 to December 2022. After data cleaning, a final dataset of 8201 EHRs was included in this study, with patient mean age of 31.79 (SD 4.63) years. Grouped by ART, there are in total 11 different ART methods, which can be generally assigned to 3 ART generations: in vitro fertilization (IVF), intracytoplasmic sperm injection (ICSI), and preimplantation genetic testing (PGT). Since the WHO has regulated the names of PGT and its subtypes, we convert all the outdated names, such as PGD (now PGT for monogenic disorders [PGT-M]), PGS (now PGT for aneuploidy [PGT-A]), to the stipulated ones. Statistically, there are 71.21% (5840/8201) IVF, including 59.6% (4885/8201) standard IVF, 8.17% (670/8201) short protocol IVF (short-time insemination), 3.48% (285/8201) IVF with donor sperm; 17.92% (1470/8201) ICSI, including 11.57% (949/8201) standard ICSI, 3.68% (302/8201) testicular sperm aspiration (TESA)+ICSI, 1.89% (155/8201) IVF+ICSI, 0.65% (53/8201) ICSI with frozen sperm, and 0.13% (11/8201) ICSI with donor sperm; 10.86% (891/8201) PGT, including 5.12% (420/8201) preimplantation genetic testing for structural rearrangements (PGT-SR), 3.76% (308/8201) PGT-A, and 1.98% (163/8201) PGT-M. For COS regimen, there are 12 categories: gonadotropin-releasing hormone antagonist fixed protocol (antagonist-fixed), luteal short-acting long protocol (luteal-short), gonadotropin-releasing hormone antagonist flexible protocol (antagonist-flex), follicular phase long-acting protocol (long-acting), progestin-primed ovarian stimulation protocol (PPOS), clomiphene citrate (CC) plus gonadotropins (CC+gonadotropin), mild stimulation protocol or direct gonadotropin protocol (mild or direct gonadotropin), luteal phase stimulation protocol (luteal-stim), clomiphene or letrozole combined with gonadotropins (CC/Letro+gonadotropin), conventional ultra-long protocol (ultra-long), modified ultra-long protocol (mod-ulong), short protocol (GnRH [gonadotropin-releasing hormone]-a short protocol; short).

### Ethical Considerations

This study is a retrospective secondary analysis of deidentified EHRs. The study protocol was reviewed and approved by the Ethics Committee of West China Second University Hospital, Sichuan University (approval 2022288). Given the retrospective design and the use of fully deidentified records, the Ethics Committee waived the requirement for additional informed consent for this secondary analysis; the original general informed consent obtained at the time of clinical care, together with the institutional policy on secondary research use of deidentified data, was determined to be sufficient.

All EHR data were fully deidentified before extraction. The deidentification procedure followed HIPAA (Health Insurance Portability and Accountability Act) Safe Harbor standards, including the removal of all direct identifiers and quasi-identifiers (eg, names, medical record numbers, contact information, and exact dates other than years of treatment). Data were stored on institutional secure servers, accessible only to authorized study personnel under the institutional data-protection policy. Outputs generated by the language models were inspected by the study team before being shown to reviewers, and any incidental identifiable strings produced by the model were redacted before expert review. No additional compensation was provided. The manuscript and supplementary materials do not contain any images, screenshots, or quotations that could identify individual patients.

### Dataset Curation

#### SFT Dataset Construction

The SFT dataset requires training pairs of prompt inputs and ground truth outputs. For the input prompt, we collated 9 fields per patient, ranging from structured baseline data to unstructured textual descriptions. The structured data included female age, menstrual cycle, weight, BMI, anti-Müllerian hormone, follicle-stimulating hormone, and infertility years. The unstructured annotations included gynecology ultrasound reports and medical history, which typically document previous conditions such as surgical history, assisted reproduction history, and male semen analysis. Because the unstructured clinical narratives (gynecology ultrasound reports and medical history) were recorded in Chinese, all narratives were translated into English using ChatGPT-4o (OpenAI) under fixed prompts that instructed the model to retain medically important information, use professional or standard medical terminology, and translate accurately and concisely; the medical-history prompt additionally specified that the translation should be produced without speculation. For the ground truth output, we designed a double-layer structure to ensure explainability: (1) a multipart clinical reasoning (chain-of-thought [CoT]), and (2) the final diagnosis and treatment plan.

However, manually annotating thousands of clinical reasoning chains was neither time-permitted nor affordable. Therefore, we generated the CoT component using an ICL approach with a diverse case boutique prompt as a few-shot prompt. This ICL-based CoT generation approach was systematically validated in our previous work [27], in which physicians confirmed that the generated reasoning chains were clinically accurate and consistent with expert-level diagnostic logic. The boutique set included 6 commonplace ART cases, with sample CoTs carefully curated by 2 expert-level physicians (these cases were excluded from our basic dataset). The resulting CoTs were structured into the following 4 aspects: diagnosis reasoning, assisted reproduction technology decision, ovarian stimulation protocol selection, and gonadotropin initiation dosing rationale. These 4 are relevant to part 2, diagnosis and treatment plan. In the second part, we set 5 final answer fields to mimic the physician’s final decision-making annotation:

- Diagnosis fields: infertility type (primary, secondary, or other) and initial differential diagnosis.
- Treatment fields: ART strategy (11 subtypes), COS regimen (12 different protocols), and the gonadotropin starting dose.

For diagnosis, infertility type judgment and initial differential diagnosis were included. Each case was labeled with 1 of the following 3 infertility categories: primary infertility, secondary infertility, or other (unclear or multifactorial cases). The initial diagnosis mainly focuses on the female side’s potential causes, and the male side is included if applicable. The ART strategy, the COS regimen, and the gonadotropin starting dose were used for the treatment plan. These three vital plans build the foundation of the following assisted reproduction and can be inferred through the comprehensive input information. The COS regimen also has 12 different protocols. In our training process, we separate the entire dataset into the training set (6559/8201, 80%), the validation set (821/8201, 10%), and the test set (821/8201, 10%)

#### Pyramid Dataset Construction

##### Overview

Before any alignment-related processing, the full curated dataset of 8201 cases was partitioned into a training set of 7380 cases (90%, same as the SFT training set and validation set) and a test set of 821 cases (10%). The 821-case test set was used exclusively for the final evaluation of all 4 alignment strategies and was never accessed during SFT training, during pyramid dataset construction, or during any subsequent DPO or GRPO training step.

To further strengthen posttraining alignment and improve the model’s discrimination on clinically ambiguous or low-frequency cases, we constructed a pyramid-style alignment dataset drawn entirely from the 7380-case training set. This dataset specifically addresses 2 limitations of the SFT baseline—its bias toward dominant treatment categories and reduced robustness in rare or clinically complex scenarios. The pyramid consists of 3 layers (general enhancement, confusion enhancement, and human enhancement), described further in this study. After SFT was completed, we performed SFT inference on the 7380 training cases to obtain model-generated responses paired with the ground-truth annotations of the same training cases. Pyramid samples were drawn from these training-set inferences. SFT inference was also performed on the 821-case test set, but only for the purpose of reporting SFT evaluation metrics; test-set inferences were never included in the pyramid dataset and were never used to construct DPO preference pairs or GRPO reward signals. The full alignment dataset was then assembled in a top-down manner into top layer, middle layer, and bottom layer.

##### Top Layer: Human Enhancement

This layer focuses on the most clinically challenging and sparsely represented cases. Two representative long-tail categories were selected: (1) IVF+ICSI, a hybrid of 2 ART generations that accounts for only 1.89% (155/8201) of SFT training data; and (2) short-protocol IVF, a complicated variant of standard IVF that is difficult even for specialists. A total of 50 cases (25 per category) were curated to maximize signal clarity and expose the model to high-value reasoning patterns rarely encountered in routine data.

##### Middle Layer: Confusion Enhancement

**Figure 2.**
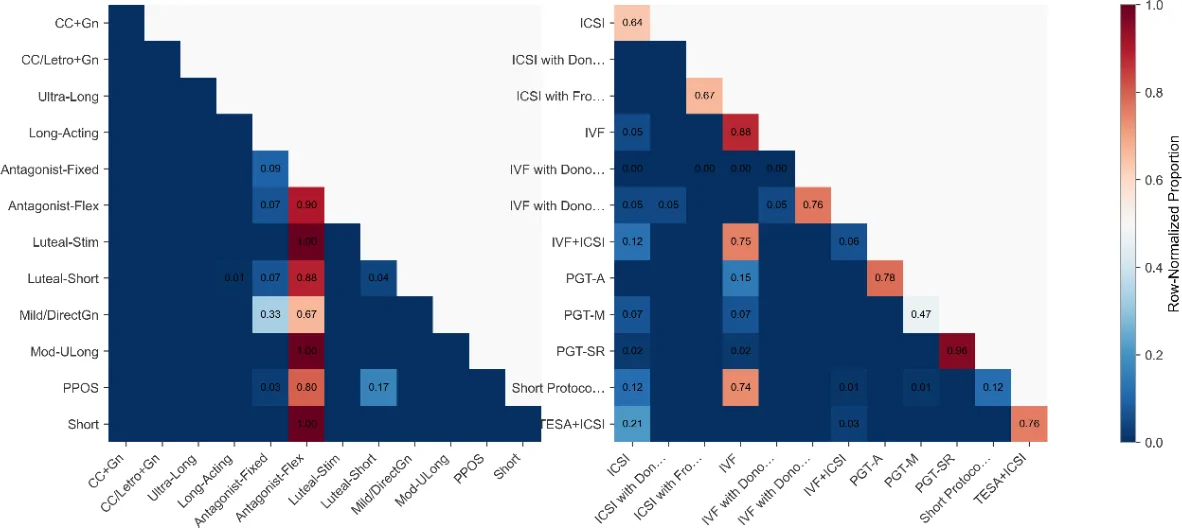
Cross-metric confusion analysis of the supervised fine-tuning baseline model on infertility decision tasks. (A) Row-normalized confusion matrix for controlled ovarian stimulation (COS) regimen selection across 12 protocol categories; (B) confusion matrix for ART strategy prediction across IVF, ICSI, and PGT. Color intensity indicating normalized frequency (e.g, blue=under-prediction, red=over-prediction). Each cell denotes the proportion of ground-truth cases assigned to a predicted category; diagonal cells represent correct predictions. These matrices reveal systematic confusion patterns, particularly the model’s tendency to over-predict dominant categories such as the Antagonist regimen and IVF. High-confusion regions identified here informed the construction of the middle “confusion enhancement” layer of the pyramid alignment dataset.

This layer focuses on systematic confusion observed in the predictions of the initial SFT model. We evaluated the outputs across both ART strategy and COS regimen, using row-normalized confusion matrices (Figure 2) to identify high-confusion regions. A total of 628 samples were extracted from categories with individual error rates greater than 10%. Particularly, poor discrimination among nonantagonist COS regimens led to the inclusion of all other regimen types, allowing this layer to fully capture the blind spots of the model.

##### Bottom Layer: General Enhancement

To avoid overfitting toward rare or confusing samples, the base layer includes a balanced mixture that includes all groups of cases. This bottom layer contains 1000 samples. At least 700 samples with 1 incorrect field and 300 completely correct samples were included across the ART strategy and COS regimen tasks. This design is to provide stable generalization support and prevent distributional skew introduced by the upper layers.

The pyramid alignment dataset contains 1678 samples in total (1666 unique training-set cases, with a small number of cases appearing in more than 1 layer). This pyramid dataset was internally split 90%:10% into alignment-training and alignment-validation subsets, which were used to train and monitor the DPO and GRPO models. The 821-case held-out test set was not part of this internal split and was never used for pyramid construction, preference-pair construction, reward computation, model selection, or alignment monitoring. We verified that the intersection between held-out test-set case identifiers and pyramid-alignment case identifiers was zero. All final metrics reported in the Results section were computed exclusively on the 821-case held-out test set.

### Modeling

To investigate how different alignment paradigms influence clinical reasoning and decision-making, we compare representative strategies built on a shared backbone. Given the clinical nature of our task, we opted not to directly fine-tune a general-purpose pretrained model, such as Qwen-2.5 (Alibaba Cloud) or LLaMA-3 (Meta). Instead, we used OpenBioLLM-8b (Saama AI Labs) [28], a domain-specific open-sourced model tailored for the medical field, which has demonstrated strong performance compared with other SOTA open-sourced models at the time of use. OpenBioLLM is built upon LLaMA-3 and has been trained on a range of medical knowledge sources. It has been adopted in several studies across clinical natural language processing and vision-language applications [29,30]. We evaluate 4 alignment paradigms—SFT, DPO, GRPO, and ICL—chosen to represent supervised, preference-based, reinforcement-based, and prompt-based alignment strategies. These 4 paradigms jointly span the major alignment families used in contemporary LLM research, enabling us to systematically assess how algorithmic optimization interacts with clinical reasoning and trust.

In this study, we aim to obtain an LLM with specific capability in infertility diagnosis and treatment planning, accompanied by the reasoning text, or CoT on each aspect. We define infertility diagnosis and treatment planning as a structured, multioutput reasoning problem. Given a patient record 𝑋 = {𝑥1, 𝑥2, 𝑥3 … , 𝑥𝑛}, which integrates both structured baseline data and unstructured clinical narratives (eg, gynecology ultrasound reports and medical history), the model 𝑓_𝜃_ is required to generate 5 interdependent clinical decisions and their corresponding reasoning chains. The task can be expressed as learning a mapping function:


(1) $$f_{\theta }:X\to (Y_{1},Y_{2},Y_{3},Y_{4},Y_{5},C)$$

where (𝑌1, 𝑌2, 𝑌3, 𝑌4, and 𝑌5) represent five structured outputs: (1) infertility type judgment, (2) initial diagnosis, (3) ART strategy, (4) COS regimen, (5) gonadotropin starting dose.

𝐶 denotes the CoT reasoning text that supports and explains each decision. The learning objective of the model is to maximize the joint likelihood of generating both the reasoning process and the final structured decisions:


(2) $$L(\theta )=\sum_{(X,Y_{1},Y_{2},Y_{3},Y_{4},Y_{5},C)\in D}\log P_{\theta }(Y_{1},Y_{2},Y_{3},Y_{4},Y_{5},C|X)$$

This provides a unified objective for multiple alignment paradigms. The model learns both to produce correct clinical outcomes and to generate transparent and clinically correct reasoning chains. We trained the models and evaluated their performance based on this objective function. A dual evaluation pipeline that includes both algorithmic metrics and human expert feedback was used. The whole process is presented in Figure 1.

### SFT

SFT is the foundation for all subsequent alignment strategies. A clinically coherent policy network pretrained with structured reasoning is formed. In this stage, the base model was trained using the training set. Each case includes a structured clinical description and an expert response for reasoning and diagnosis, as previously mentioned in the data section. Training was performed with low-rank adaptation (LoRA; learning rate=3e-5, batch size=4) for 10 epochs on a single A100 GPU. The resulting model serves as the reference policy for the subsequent stages. The number of training epochs was selected based on validation-set loss plateau. Training loss converged after epoch 7, and validation loss showed no further improvement after epoch 10; the checkpoint at epoch 10 was retained for downstream alignment experiments.

### DPO

This variant focuses on aligning model reasoning with expert preferences by directly optimizing the token-level log-likelihood contrast between preferred and dispreferred responses. Direct DPO is a critic-free reinforcement learning method that has recently emerged from the RLHF paradigm [6]. It provides a relatively simple and stable alternative to reward-model–based offline approaches for aligning language models with human preferences and has shown promising results in reducing undesirable behaviors in baseline models. Unlike traditional RLHF methods that rely on learning a separate reward model or value function, DPO directly optimizes the policy by contrasting preferred and dispreferred responses. Specifically, each training data point consists of a prompt *x*, a preferred response 𝑦_𝑤_, and a less-preferred response 𝑦_ι_. Given the prompt *x*, the DPO loss encourages the policy model to assign a higher likelihood to 𝑦_𝑤_ over 𝑦_ι_, thereby aligning the model outputs more closely with human preferences. The DPO loss function is formulated as:


(3) $$L_{DPO}(\pi_{\theta };\pi_{ref})=-E_{(x,y_{w},y_{l})∼D}\left[\log\sigma \left(\beta \left[\log\frac{\pi_{\theta }(y_{w}|x)}{\pi_{ref}(y_{w}|x)}-\log\frac{\pi_{\theta }(y_{l}|x)}{\pi_{ref}(y_{l}|x)}\right]\right)\right]$$

𝜋_𝜃_ is the current policy model, σ is the sigmoid function, and β is a temperature parameter that controls the sharpness of the preference. This loss function assigns equal weight to all tokens in each response. By doing so, we aim to align the reasoning process and final diagnosis to the human level. Compared with the correct final answer, this setup helps the model to predict the correct outcome, as well as generating a clinically sound CoT. In the context of our study, where both the intermediate reasoning (eg, identifying relevant clinical clues) and the final decision (eg, treatment plan) are critical, such token-level uniform supervision helps ensure that the model learns to reflect expert-like logic across the entire response, rather than merely optimizing for the final output token. In our experiments, we use the SFT model described in SFT section as the reference policy. DPO was conducted on a single A100 GPU with *β*=.3, learning rate=3e-7, and batch size=8 for 1 epoch. Single-epoch DPO follows the typical protocol and is consistent with broader RLHF/DPO practice, in which multiepoch training over a fixed preference dataset typically leads to overoptimization on the preference signal and policy collapse away from the reference SFT model. To directly rule out undertraining as a confounder of the relative DPO underperformance, we additionally trained DPO under 2 longer configurations on the same preference dataset and identical hyperparameters: a 2-epoch configuration and a 10-epoch configuration with early stopping on validation loss. Field-level accuracy on the held-out test set degraded sharply rather than improved as training proceeded. Infertility-type accuracy fell from 92.2% (1 epoch) to 72% (2 epoch) to 53.6% (10 epoch with early stopping). COS regimen accuracy fell from 60.9% to 38% to 18%. ART strategy accuracy fell from 75.03% to 12% to 9.4%. Gonadotropin starting-dose mean absolute error (MAE) rose from 45.12 IU to 55.50 IU to 58.45 IU. This pattern is consistent with the well-documented DPO collapse phenomenon, and the 1-epoch checkpoint was retained as the reported configuration.

### GRPO

This variant leverages reinforcement-based relative optimization to enhance decision consistency while eliminating the computational overhead of value-function training. GRPO is an evolutionary variant of proximal policy optimization (PPO) [31]. For PPO, its advantage is computed by applying generalized advantage estimate [32], based on the rewards and a learned value function. Consequently, a value function needs to be trained alongside the policy model and using a per-token KL penalty from the reference model to mitigate overoptimization of the reward model. As the value function is involved in the training, it is typically another model of comparable size to the policy model, bringing a gargantuan computational burden. While for GRPO, it obviates an additional value function for advantage computation and instead uses the reward average of multiple outputs sampled from the same question as the baseline. More specifically, for each question *q*, GRPO samples a group of outputs {𝑜1, 𝑜2, … , 𝑜𝐺} from the old policy 𝜋_𝜃_ and then optimizes the policy model by maximizing the following objective:


(4) $$\begin{array}{l} J_{GRPO}(\theta )=E_{q∼P(Q),\;\{o_{i}{\}}_{i=1}^{G}∼\pi_{\theta_{old}}}\left[\frac{1}{G}\sum_{i=1}^{G}\frac{1}{|o_{i}|}\sum_{t=1}^{|o_{i}|}\left\{\min\;\left[\frac{\pi_{\theta }(o_{i,t}|q,o_{i,§lt;t})}{\pi_{\theta_{old}}(o_{i,t}|q,o_{i,§lt;t})}{\hat{A}}_{i,t},\;\;\mathrm{clip}\;\left(\frac{\pi_{\theta }(o_{i,t}|q,o_{i,§lt;t})}{\pi_{\theta_{old}}(o_{i,t}|q,o_{i,§lt;t})},1-\varepsilon ,1+\varepsilon \right){\hat{A}}_{i,t}\right]-\beta D_{KL}\;\left[\pi_{\theta }\;\|\;\pi_{ref}\right]\right\}\right] \end{array}$$

where ε and β are hyperparameters, and 𝐴̂ _{𝑖,𝑡}_ is the advantage calculated based on relative rewards of the outputs inside each group only, which will be detailed in the following subsections. GRPO offers a compelling solution for medical domains where training a value function is often impractical due to limited data, and where structured, interpretable supervision is essential for aligning model behavior with expert expectations.

Reward design follows the common practices [8] by using the accuracy reward, aimed at achieving the “algorithm alignment.” Since we have 4 structured final answer fields, except for the initial diagnosis, which comprises long sentences, we used a combined final reward to aggregate 4 separate rewards:


(5) $$r_{i}=\lambda_{IT}\;r_{i}^{IT}+\lambda_{COS}\;r_{i}^{COS}+\lambda_{Gn}\;r_{i}^{Gn}+\lambda_{ART}\;r_{i}^{ART}.$$

The weight for each field was set to:


(6) $$\lambda_{IT}=0.2,\;\lambda_{COS}=0.3,\;\lambda_{Gn}=0.2,\;\lambda_{ART}=0.3.$$

The weighting coefficients were chosen on clinical grounds rather than tuned by formal hyperparameter search. COS regimen and ART strategy directly determine the treatment course and downstream patient management and were therefore assigned the higher weight (0.3 each). Infertility type is largely derivable from structured baseline information and was assigned a lower weight (0.2). The gonadotropin starting dose is a continuous numerical decision and is additionally handled by a graded reward described below, so its weight in the combined accuracy reward was set to the same lower value (0.2). The 4 weights sum to 1.0. We did not perform a full reward-weight ablation at training time within the present study. Additionally, as the gonadotropin dose is a numerical answer, to evaluate the predicted gonadotropin starting dose, we designed a graded reward function that reflects real-world clinical practices. In dealing with clinical ovarian stimulation protocols, the gonadotropin dose is typically adjusted in increments of 25 IU. Thus, a deviation of 25 IU is clinically acceptable. Therefore, we assign:


(7) $$r_{Gn}=\left\{ \begin{array}{ll} 1.0, & \text{if }|\hat{y}-y|\leq 25,\\ 0.5, & \text{if }25§lt;|\hat{y}-y|\leq 50,\\ 0.0, & \text{otherwise.} \end{array}\right.$$

This reward strategy helps the model to approximate the clinically preferred range, even if the value is not exactly matched. It reflects the tolerance that physicians often exhibit during dose adjustment in clinical practice.

GRPO training configuration: GRPO was implemented using the verl framework with the trainer configured for GRPO advantage estimation. The actor was initialized from the SFT checkpoint and fine-tuned with LoRA adapters (rank=64, α=32). Training used a learning rate of 3e-5 for 5 epochs (best at 1 epoch), with a training batch size of 16, and the PPO clipping ratio ε at the verl default value of 0.2. The KL loss coefficient was 0.001 with the low-variance KL estimator, and the rollout group size was n=5 per prompt. Training was conducted on 1 node with 4 NVIDIA A100 GPUs.

### ICL: Guideline-Based Prompt Alignment

The primary purpose of using ICL is to enhance reasoning generalization through guideline-based prompts. ICL is a training-free post-alignment approach commonly applied to pretrained models. Conventional few-shot ICL relies on instance-level examples. Our variant repurposes clinical heuristic maps into textual guidance blocks. These guidelines are oriented from physicians’ decision flowcharts and are intended to translate structured expert reasoning paths (eg, stimulation protocol selection) into natural-language rules embedded in the prompt. This design transforms medical knowledge into interpretable prompt-based supervision, allowing the model to internalize domain heuristics without additional parameter updates. We proposed 2 guidelines on the ART strategy and COS regimen in this variant. The SFT model is retained as the core inference framework. This is mainly due to its domain-specific alignment, particularly its ability to maintain good output consistency and clinical reasoning. Two guideline blocks are inserted into the prompt for each case (refer to Apendix S1 in Multimedia Appendix 1 for the full guideline blocks used in the ICL prompt). Additionally, we prepend the following instruction:

**“DO NOT USE THE GUIDELINE UNLESS YOU ARE NOT SURE ABOUT YOUR ANSWER.”**

This design helps the model to rely mainly on its internalized reasoning ability and consult the guideline only when uncertain. In this way, the instructional ICL serves as a lightweight and interpretable alternative to gradient-based alignment, effectively simulating real-world physician behavior, where decisions are primarily experience-driven, with reference to protocols only when ambiguity arises. We acknowledge that this gated prompt design relies on the language model’s ability to assess its own epistemic uncertainty, a capability that contemporary LLMs are known to calibrate poorly. We intentionally retained this gating because it mirrors real-world physician behavior in which decisions are primarily experience-driven and external guidelines are consulted only when ambiguity arises. We did not evaluate alternative prompt configurations (eg, unconditional guideline conditioning, retrieval-augmented prompting, or chain-of-verification approaches) in this study. The ICL results reported here therefore characterize this specific gated prompt configuration as we implemented it and should not be read as a general claim about prompt-based alignment as a paradigm.

### Dual-Evaluation Protocol

We used a dual-layer evaluation framework to assess both quantitative task performance and qualitative clinical quality. This framework integrates (1) automatic field-level metrics and (2) blinded independent expert review. This design ensures comprehensive examination of both algorithmic accuracy and clinician-perceived interpretability.

### Automatic Evaluation

Model outputs were evaluated using standard metrics for structured clinical fields. Specifically, we compute accuracy and macro-*F*_1_ for categorical predictions, including infertility type, ART strategy, and COS regimen. Specifically, the macro-*F*_1_ is calculated with the following formula:


(8) $$Macro\;F1=\frac{1}{K}\sum_{i=1}^{K}F1_{i}$$

Where 𝐾 denotes the total number of categories, 𝐹_1𝑖_ is the 𝐹_1_ score of the 𝑖 category.

Because the initial diagnosis field exhibits high variability and paraphrasing, exact word-by-word matching is infeasible and unreliable. Therefore, an auxiliary LLM was used as a semantic judge to determine whether the generated diagnosis semantically entailed the ground-truth diagnosis. We report the precision of containing at least one true diagnosis or all of the ground truth. For the numerical field of gonadotropin starting dose, prediction error was quantified using MAE. Together, these metrics assess the model’s structured decision-making performance but do not capture the quality of its reasoning process.

### Doctor-in-the-Loop Evaluation

To evaluate clinical reasoning and decision-making quality beyond quantitative metrics, we conducted a domain-expert assessment. This evaluation addressed two core questions:

1. Is there a possible decoupling between “algorithmic alignment” and “clinical alignment”? Specifically, does aggressive outcome optimization come at the hidden cost of sacrificing reasoning quality and therapeutic feasibility?
2. How well do the aligned models simulate expert-level performance?

Experts rated each output on 4 clinical dimensions:

- Clinical reasoning capability: whether the model is a medically sound thought process.
- Diagnosis accuracy: whether the inferred infertility type and the Initial diagnosis are correct.
- Treatment feasibility: whether the recommended treatment plan is appropriate and consistent.
- Hallucination: whether the reasoning process contains irrelevant or wrong information.

These dimensions are a relatively comprehensive subjective evaluation. Each output used a 5-point Likert scale (1=poor, 5=excellent). The fourth dimension, Hallucination, was evaluated as a binary judgment (1=yes, 0=no) rather than on the Likert scale. We compare the outputs of the SFT model and the best-performing postalignment model for each evaluation case. Additionally, to further benchmark model performance against clinical standards, a third response from the ground truth was included, which is used to represent the expert-level response. The graders are then asked to select the overall best response. For the 3-way best-response task, all candidate responses were presented in a standardized response format containing a reasoning section and a final treatment-plan section. In the reference response, the diagnosis and treatment plan were the authentic physician-charted ground truth. The accompanying reasoning narrative was generated by ChatGPT-4o from that charted decision, using the same specialist-curated CoT prompt applied during SFT dataset construction, and was subsequently reviewed and edited by 2 reproductive medicine specialists. The clinical decision was therefore human-authored, whereas the reasoning narrative was model-drafted and physician-verified. To reduce source-recognition bias, response labels, model identifiers, and original chart-note markers were removed, and the 3 candidate responses were randomized before expert review. In a small number of cases reviewers indicated a tie between 2 or 3 responses (eg, both SFT and the charted plan rated as equivalently best); for 2-way ties, each tied option received 0.5; for 3-way ties, each option received 0.33. The per-rater fractional preferences were then averaged across the 2 reviewers (Y Long and TT) and the resulting proportions are reported.

All responses are blindly evaluated. Model identifiers and response labels were removed before review. However, because the 3-way comparison included a physician-certified reference response derived from charted care, residual source-recognition bias and partial unblinding cannot be fully excluded. Therefore, this comparison should be interpreted as a standardized best-response preference task rather than a fully clean blinded comparison between models and physician decision-making. The graders are unaware of the matching relationship to reduce bias. Each case is independently reviewed by 2 experienced physicians (Y Long and TT) in reproductive medicine. Considering the limited time available for physicians, we selected 100 representative cases as the evaluation set. The expert review should be interpreted as an exploratory clinical-quality assessment rather than a definitive clinical validation study.

Interrater reliability was quantified using 5 complementary metrics, because raw Cohen κ is known to be unreliable when marginal rating distributions are highly skewed, a situation referred to as the κ paradox. We report raw observed agreement (P_o), agreement within one Likert point (P_o±1), quadratic-weighted Cohen κ, prevalence-adjusted bias-adjusted kappa (PABAK), and Gwet AC1 coefficient. The latter 2 metrics are designed to be robust to skewed marginal distributions. All metrics are reported per Likert dimension and pooled across the 3 dimensions evaluated by both reviewers.

To assess whether the custom graded gonadotropin starting-dose reward and the per-field correctness signals propagated into the subjective treatment-feasibility ratings, we conducted a post hoc correlation analysis between the per-case absolute gonadotropin dose error, per-case COS regimen correctness, per-case ART treatment correctness, and the per-case mean feasibility score (averaged across the two raters) for both SFT and GRPO outputs. Pearson and Spearman correlations and case-stratified mean comparisons (dose-correct vs dose-incorrect, and by tertile of dose error) are reported in the Results section.

All outputs previously flagged as hallucinated were subsequently reviewed and categorized into low-severity, moderate-severity, or potentially unsafe hallucinations according to whether the unsupported content could alter diagnosis, ART strategy, COS regimen, gonadotropin dosing, or patient safety. All hallucination-positive rater–output judgments were subsequently reviewed by the same 2 reproductive medicine specialists who performed the original expert evaluation. The specialists remained blinded to model identity, and disagreements were resolved by consensus.

### Statistical Analysis

For automatic evaluation, accuracy, macro-*F*_1_, and MAE were reported as point estimates across all structured decision fields, with 95% CIs computed via nonparametric bootstrap resampling (n=2000 iterations stratified by case). The point estimates themselves were not subjected to inferential significance testing because they are deterministic functions of the model outputs on a fixed held-out test set. To assess whether differences in per-case classification correctness between models were unlikely to arise from sampling variation, we additionally performed pairwise comparisons on categorical fields (infertility type, ART strategy, and COS regimen) using the exact McNemar test on the per-case correctness indicator. Holm-Bonferroni correction was applied across the 9 pairwise model comparisons. Both raw and Holm-adjusted *P* values are reported.

For the blinded independent expert review, three clinical dimensions (clinical reasoning capability, diagnostic accuracy, and treatment feasibility) were scored on a 5-point Likert scale. Hallucination was evaluated separately as a binary per-output judgment and summarized as a rate. Likert ratings are ordinal; we therefore report medians and IQRs as the primary descriptive statistics, with means and SDs reported alongside for comparability with previous work. Pairwise comparisons of expert-rated dimensions between SFT and GRPO used Wilcoxon signed-rank tests on the per-case mean of the 2 raters, with Holm-Bonferroni correction across the 3 Likert dimensions. Effect sizes are reported as paired Cohen *d*, with the limitations of treating ordinal Likert data as continuous explicitly noted in the Limitations section. Interrater reliability was quantified using multiple complementary metrics, including raw observed agreement, within-one-point agreement, quadratic-weighted Cohen κ, PABAK, and Gwet AC1 coefficient, because raw Cohen κ is uninformative when marginal rating distributions are highly skewed. For clarity, differences between model accuracies are reported as absolute percentage-point differences, with relative percent changes provided in parentheses when useful.

## Results

### Automatic Metrics Performance

Reinforcement-based alignment achieves superior algorithmic precision across structured decision layers. GRPO establishes a higher automatic field-level performance over SFT (baseline model) in objective outcome metrics. Table 1 summarizes the field-level performance of the 4 models. GRPO (the SFT model trained by GRPO) achieves relatively the best overall results in the fields of Infertility type, ART strategy, and COS regimen. Individually, GRPO has the highest accuracy of 92.57% of infertility type judgment, 76.49% of ART strategy selection, and 89.4% of initial diagnosis partial match, and 20.32% of exact match. Compared with the base SFT model, GRPO improves ART strategy selection accuracy by 2.68% points, though this did not survive correction for multiple comparisons (McNemar p_raw=0.020, p_Holm=0.118). As for the unstructured Initial diagnosis task, GRPO also demonstrates clear enhancement, with a 1.95 percentage points higher partial match rate and a 4.24% points increase in exact matches. Overall, GRPO achieves the highest average accuracy (77.14%) and macro-*F*_1_ score (50.64%). For other models, DPO shows moderate gains in ART strategy choice (accuracy+1.22% vs SFT), which indicates the effectiveness of the preference alignment training strategy. However, its instability is reflected in the initial diagnosis task. DPO showed a substantial decline in initial-diagnosis performance, with partial match decreasing by 46.16% points relative to SFT (87.45% to 41.29%) and exact match decreasing by 11.45% points (16.08%-4.63%). While for the last model, although built on the same SFT backbone, ICL failed in nearly all metrics, with only a marginal improvement in ART strategy selection (accuracy+0.95%). Notably, all models struggle with the COS regimen prediction. This may be due to the possible inherently ambiguous category boundaries. As the overall supreme model in the other field, GRPO records a slight decrease in accuracy (−1.34 percentage points vs SFT) and *F*_1_ (−0.88 vs SFT), while DPO and ICL exhibit a larger accuracy drop (−2.80%, −4.26% vs SFT), despite a minor *F*_1_ improvement by DPO (+0.56 vs SFT).

**Table 1.** Field-level performance comparison of SFT^a^, ICL^b^, DPO^c^, and GRPO^d^ models on infertility-related decision tasks. Results are reported for 5 evaluation fields: infertility type classification, ART^e^ strategy selection, COS^f^ regimen prediction, initial diagnosis (partial and exact match), and gonadotropin starting dose (IU). Overall, GRPO demonstrates the highest accuracy and F1 score.

Model	Infertility type		ART strategy		COS regimen		Average		Initial diagnosis		Gonadotropin starting dose (IU)
	Accuracy (95% CI)	Macro *F*_1_-score	Accuracy (95% CI)	Macro *F*_1_-score	Accuracy (95% CI)	Macro *F*_1_-score	Accuracy	Macro *F*_1_-score	Partial	Exact	MAE^g^ (95% CI)
SFT	92.57 (90.7‐94.4)	92.18^h^	73.81 (70.8‐76.7)	49.18	63.70 (60.4‐66.8)^h^	7.75	76.69	49.70	87.45	16.08	43.88 (40.7‐47.0)^h^
ICL	90.83 (88.4‐92.4)	89.88	74.76 (72.1‐77.8)	33.31	59.44 (56.0‐62.6)	5.89	75.01	43.03	87.21	16.09	48.38 (45.2‐51.6)
DPO	92.20 (90.3‐94.0)	91.33	75.03 (72.4‐78.3)	46.62	60.90 (57.6‐64.1)	8.31^h^	76.04	48.75	41.29	4.63	45.12 (42.0‐51.6)
GRPO	92.57 (90.6‐94.3)^h^	92.05	76.49 (73.9‐79.7)^h^	52.99	62.36 (59.0‐65.4)	6.87	77.14^h^	50.64^h^	89.40^h^	20.32^h^	44.94 (41.7‐48.2)

a SFT: supervised fine-tuning.

b ICL: in-context learning.

c DPO: direct preference optimization.

d GRPO: group relative policy optimization.

e ART: assisted reproductive technology.

f COS: controlled ovarian stimulation.

g MAE: mean absolute error.

h highlights the best performance in each metric.

### Doctor-in-the-Loop Evaluation

Our results suggest that algorithmic gains may not automatically lead to clinical trust. In contrast, the evaluation results show that the reasoning clarity and regimen feasibility of the baseline output are directionally higher rated by practitioners compared to others. We used human feedback from two reproductive experts to evaluate the models’ clinical performance in real-world workflow. We selected the baseline model (SFT) and the posttraining model (GRPO), which achieved the highest automatic scores. Each model’s output was scored on 4 primary dimensions: diagnosis accuracy, clinical reasoning capability (reasoning), treatment plan clinical feasibility (feasibility), and hallucination. The rating distributions across the 2 reviewers, the 3 Likert dimensions, and the 2 models were strongly concentrated at the upper end of the scale (62.3% of ratings were 4, 23.8% were 5, 13.8% were 3, and fewer than 0.3% were 1 or 2). Under this regime raw Cohen κ is uninformative because chance agreement is already very high. The 2 reviewers gave the same Likert score on 41% of pooled ratings and agreed within one Likert point on 93.7% of pooled ratings. Quadratic-weighted Cohen κ was 0.05 pooled (range 0.03 to 0.06 across dimensions). PABAK was 0.26 pooled (accuracy=0.30, reasoning=0.28, and feasibility=0.21). Gwet AC1 was 0.32 pooled (accuracy=0.35, reasoning=0.33, and feasibility=0.28). Both kappa-paradox-robust metrics fall in the fair-to-moderate range on the Landis and Koch scale, consistent with the high within-one-point agreement.

The results of this expert scoring (Multimedia Appendix 2) revealed a notable discrepancy with the findings from the automated metrics. Median scores were 4.00 (IQR 4.00‐4.50) for SFT versus 4.00 (IQR 3.50‐4.50) for GRPO on diagnostic accuracy, 4.00 (IQR 4.00‐4.50) for SFT versus 4.00 (IQR 4.00‐4.50) for GRPO on reasoning capability, and 4.50 (IQR 4.00‐4.50) for SFT versus 4.00 (IQR 4.00‐4.50) for GRPO on treatment feasibility. The corresponding means were 4.04 (SD 0.42) vs 3.99 (SD 0.45), 4.17 (SD 0.42) vs 4.08 (SD 0.42), and 4.21 (SD 0.39) vs 4.09 (SD 0.42). Pairwise comparisons used Wilcoxon signed-rank tests on the per-case mean of the 2 raters with Holm-Bonferroni correction across the 3 dimensions; Holm-adjusted *P* values were .21 for diagnostic accuracy, .07 for reasoning capability, and .06 for treatment feasibility. Nonparametric (rank-biserial) effect sizes were 0.21, 0.30, and 0.33 respectively (small to small-to-moderate by Cohen conventions); the corresponding paired Cohen *d* values, presented for comparison with previous work and under the explicit assumption that Likert data are treated as continuous, were +0.13, +0.22, and +0.25.

Despite GRPO showed stronger overall automatic performance than SFT across several fields, the physician ratings suggested an opposite directional trend. The SFT model received directionally higher ratings for reasoning capability and treatment feasibility (mean SFT 4.17, SD 0.42; mean GRPO 4.08, SD 0.42; Cohen *d*=0.22, mean difference 95% CI 0.005-0.170; *P*=.03, adjusted *P*=.07) and clinical feasibility (mean SFT 4.21, SD 0.39; mean GRPO 4.09, SD 0.42; Cohen *d*=0.25, mean difference 95% CI 0.025-0.210; *P*=.02, adjusted *P*=.06). There were no significant differences observed in diagnosis accuracy between the 2 models. For the additional dimension, hallucination, GRPO was rated to have a lower rate (15%) compared with SFT (18.5%). Among outputs flagged as hallucinated, most hallucinations were low-severity extraneous statements rather than safety-critical treatment errors. Specifically, 82.1% (55/67) were classified as low severity, 3% (2/67) as moderate severity, and 14.9% (10/67) as potentially unsafe. Low severity denotes a benign, clinically inert fabrication that does not alter the infertility diagnosis, ART indication, stimulation protocol, or dosing (eg, an invented “breast cyst”). Moderate severity denotes a false but noncommunicable condition or history that could prompt unnecessary work-up, referral, or patient anxiety without directly dictating harmful treatment (eg, recasting a single biochemical pregnancy as “recurrent pregnancy loss”). Potentially unsafe denotes a fabrication that could cause real harm if acted upon, which may trigger unnecessary treatment, partner notification, biosafety measures, stigma, or delay of time-sensitive care. This severity distribution explains why hallucination flags could coexist with high global Likert ratings: the Likert dimensions captured overall reasoning coherence and treatment feasibility, whereas the binary hallucination item captured any unsupported content, including low-impact extraneous information.

Our results suggest a possible trade-off. The optimization for outcome token-level precision may be the result of sacrificing process transparency and output coherence. This indicates an “alignment paradox” phenomenon. The pursuit of higher benchmark scores may lead to a negative result of a mismatch between model performance and physician expectations.

To evaluate whether the custom graded gonadotropin dose reward and the per-field correctness signals propagated into the subjective treatment-feasibility ratings, we computed the per-case association between automatic correctness and physician-rated feasibility on the same 100-case evaluation sample. The absolute gonadotropin starting-dose error was not meaningfully correlated with mean physician-rated treatment feasibility for either model (SFT: Pearson *r*=−0.11, *P*=.30; Spearman ρ=−0.09, *P*=.40; GRPO: Pearson r=+0.09, *P*=.39; Spearman ρ=+0.08, *P*=.41). Stratified comparison gave the same conclusion: mean feasibility on cases for which gonadotropin dose was exactly correct versus cases for which it was incorrect was 4.23 versus 4.19 for SFT and 4.07 versus 4.10 for GRPO (absolute differences <0.05 on a 5-point scale, with nonmonotonic patterns across error tertiles). COS regimen correctness and ART treatment correctness were similarly weak drivers of feasibility scores (absolute differences ≤0.07 between correct and incorrect subgroups for both models). These results indicate that the 2 reproductive medicine specialists rated treatment feasibility based on the overall coherence and clinical sensibility of the treatment plan as a whole rather than on the correctness of any single structured decision field. The custom graded gonadotropin dose reward used during training therefore did not propagate a confounding signal into the physician-rated feasibility comparison.

### Winning Rate

In addition to the process-level ratings, we included an overall best-response question, which asks the experts to blindly identify the best response from three responses, including 2 scored responses by models and a third response representing the physician-certified reference response. As illustrated in subpart (B) in Multimedia Appendix 2, physicians selected the output by the SFT model in more than half of the cases (51.2%, 95% CI 44.2%-58.1%), higher than the GRPO outputs (26.2%, 95% CI 20.1%-32.3%) and the original charted plans (22.6%, 95% CI 16.9%-28.5%) in this structured preference task. This result should be interpreted as a preference within the review format rather than as evidence that model-generated decisions are clinically superior to physician decision-making. When the 3-way preference is reduced to pairwise comparisons, SFT was selected over GRPO in 66.2% of head-to-head matchups (Holm-adjusted *P*<.001) and over the original physician-charted plan in 69.3% (Holm-adjusted *P*<.001), while GRPO did not differ significantly from the charted plan (53.6%, Holm-adjusted *P*=.48). These results should be interpreted cautiously, given the substantial between-rater variability: one evaluator showed a strong SFT preference (77.5% SFT over GRPO) while the other showed a more balanced distribution (54% vs 46%). This preference for model outputs over charted plans may have been partially influenced by the well-structured and completeness of model responses, which charted plans may not contain. The SFT-versus-GRPO contrast within this comparison is less subject to this consideration because both model outputs include reasoning chains generated directly by models. Specifically, it shows the same trend as the results shown in human evaluations. However, physicians are inclined to the baseline response for its reasoning clarity and decision coherence. In essence, interpretability and accuracy may be decoupled through optimization, which elevated benchmark scores but eroded the coherence that is essential for clinical trust.

Collectively, these three layers of evaluation above interpret the possible nature of the “alignment paradox.” Automatic metrics illustrate the effectiveness of reinforcement learning–based alignment in maximizing token-level outcome precision. At the same time, physicians’ ratings suggest that the simplest baseline results in the highest level of reasoning accuracy. Our blinded winning rates comparison indicates that although both paradigms have better consistency than humans, the mechanisms used to achieve this are fundamentally different. GRPO’s stronger automatic performance and directionally lower clinician ratings suggest that the current optimization goals divide “accuracy” from “logic.” As a result, the consistency between the process and the final answers may contribute to clinical trust.

### Subgroup Analysis

In high-stakes fields such as clinical question answering, performance consistency across clinical categories is as important as overall performance. Thus, how these methods influence the model output under various clinical contexts is critical. We evaluated model behavior across major ART categories (Multimedia Appendix 3). The current ART is evaluated in three main categories: IVF, ICSI, and PGT. For the PGT category, GRPO achieved a higher *F*_1_ score than SFT (SFT=0.921; GRPO=0.934). For IVF, GRPO and DPO yielded *F*_1_ scores comparable to and slightly higher than SFT (SFT=0.924; GRPO=0.926; DPO=0.925). By contrast, in the ICSI category, GRPO and DPO yielded lower *F*_1_ scores than SFT (SFT=0.721; GRPO=0.687; DPO=0.709).

**Figure 3.**
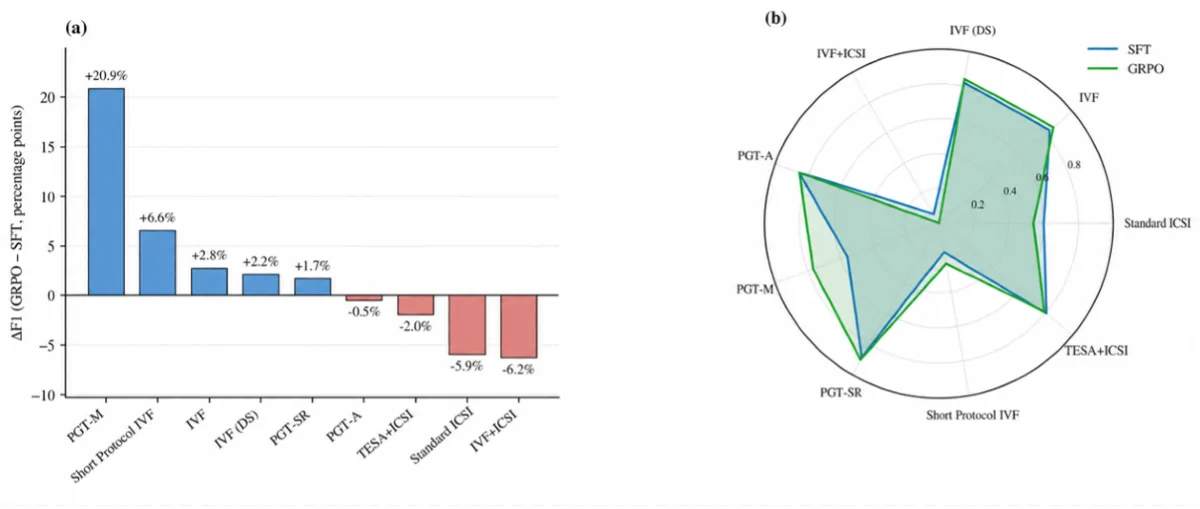
Differences between group relative policy optimization (GRPO) and supervised fine-tuning (SFT) in subgroups. Field-level differences between GRPO and SFT across detailed assisted reproductive technology (ART) subtypes in the held-out test set. (A) Per-subtype *F*_1_ difference (GRPO−SFT, percentage points) across ART subtypes. Positive values indicate improvement under GRPO. Subgroups with fewer than 10 cases are excluded and reported descriptively only. (B) Radar plot comparing subtype-level *F*_1_ scores between SFT and GRPO.

To examine the origins of these trends, we further conducted a fine-grained analysis of more detailed ART subtypes (Table 2). We used the baseline SFT and GRPO for comparison. The differences between the 2 models are illustrated in Figure 3. GRPO has poorer performance across all ICSI-related subgroups. Among subgroups with at least 10 cases, the largest *F*_1_ decrease occurred in IVF+ ICSI (from 6.2% to 0%, a decrease of 6.2% points; n=16), followed by standard ICSI (a decrease of 5.93% points). GRPO produces better gains in complex PGT subtypes, for example, PGT-M (+20.9%) and PGT-SR (+1.7%). A similar pattern was noticed for IVF subtypes. GRPO performs better in Short Protocol IVF (+6.5%) and has a moderate gain in standard IVF (+2.8%). However, we would like to acknowledge that the lower performance in ICSI with frozen sperm or donor sperm should be interpreted carefully, as the sample sizes were limited (n=3 and n=1, respectively). Subtypes with n <10 were excluded from comparative inference and are reported descriptively only.

Our findings map a detailed influence pattern of alignment in infertility medicine. While GRPO performs well for rare and complex problems (eg, PGT) through targeted reinforcement, its capability for intermediate categories like ICSI is very limited. Thus, to design trustworthy medical LLMs, it is necessary to move beyond simple data balancing to implementing adaptive fair rewards. The optimization of rare cases should not lead to decreased performance of the decision boundaries that are essential for routine care.

**Table 2.** ART^a^ subtype performance. ART subtype-level performance of the SFT^b^ baseline and the GRPO^c^-aligned model on the held-out test set. Per-subtype precision, recall, and *F*_1_-score (percentages) are reported alongside test-set sample size. Subtypes with n<10 are excluded and should be interpreted descriptively only.

ART	N	SFT			GRPO		
		Precision, % (95% CI)	Recall, % (95% CI)	*F*_1_-score	Precision, % (95% CI)	Recall, % (95% CI)	*F*_1_-score
IVF^d^	483	79.9 (76.3‐83.1)	86.5 (83.2‐89.3)	83.1	80.7 (77.2‐83.8)	91.7 (88.9‐93.9)	85.9
Standard ICSI^e^	96	57.0 (47.5‐66.0)	63.5 (53.6‐72.5)	60.1	54.2 (44.2‐63.8)	54.2 (44.2‐63.8)	54.2
Short Protocol IVF	76	30.0 (16.7‐47.9)	11.8 (6.4‐21.0)	17.0	46.2 (28.8‐64.5)	15.8 (9.3‐25.6)	23.5
PGT-SR^f^	49	83.6 (71.7‐91.1)	93.9 (83.5‐97.9)	88.5	85.2 (73.4‐92.3)	95.8 (86.0‐98.8)	90.2
TESA^g^+ICSI	33	86.2 (69.4‐94.5)	75.8 (59.0‐87.2)	80.6	85.7 (68.5‐94.3)	72.7 (55.8‐84.9)	78.7
PGT-A^h^	27	95.5 (78.2‐99.2)	77.8 (59.2‐89.4)	85.7	88.5 (71.0‐96.0)	82.1 (64.4‐92.1)	85.2
IVF with donor sperm	21	88.9 (67.2‐96.9)	76.2 (54.9‐89.4)	82.1	94.1 (73.0‐99.0)	76.2 (54.9‐89.4)	84.2
PGT-M^i^	16	77.8 (45.3‐93.7)	43.8 (23.1‐66.8)	56.0	100.0 (72.2‐100.0)	62.5 (38.6‐81.5)	76.9
IVF+ICSI	16	6.2 (1.1‐28.3)	6.2 (1.1‐28.3)	6.2	0.0 (0.0‐29.9)	0.0 (0.0‐19.4)	0.0

a ART: assisted reproductive technology.

b SFT: supervised fine-tuning.

c GRPO: group relative policy optimization.

d IVF: in vitro fertilization.

e ICSI: intracytoplasmic sperm injection.

f PGT-SR: preimplantation genetic testing for structural rearrangements.

g TESA: testicular sperm aspiration.

h PGT-A: preimplantation genetic testing for aneuploidy.

i PGT-M: preimplantation genetic testing for monogenic disorders.

## Discussion

### Principal Findings

Our principal finding is that, in this single-center retrospective evaluation of mainstream alignment paradigms for medical LLMs in ART decision support, automatic field-level accuracy and physician-rated clinical quality suggested a possible decoupling. GRPO consistently produced the highest automatic accuracy across structured decision fields; yet, 2 blinded independent expert reviewers (Y Long and TT) had marginally higher ratings of the conservative SFT baseline on reasoning capability and treatment feasibility, with no significant difference in diagnostic accuracy. We refer to this decoupling as the alignment paradox: posttraining that improves token-level outcome metrics may simultaneously degrade the process-level reasoning that clinicians use to decide whether a model output is trustworthy. The alignment paradox interpretation does not rest on any single source of evidence. Three measurements with different statistical assumptions and different vulnerabilities converge on the same direction. First, 2 blinded reviewers (Y Long and TT) rated the SFT baseline higher than GRPO on reasoning capability and treatment feasibility, with small paired effect sizes (Cohen *d*=+0.22 and+0.25) and Holm-adjusted Wilcoxon *P* values of .07 and .06. Second, in the discrete three-way best-response preference (SFT 51.2% vs GRPO 26.2% vs original charted plan 22.6%), reviewers selected SFT substantially more often than GRPO; this comparison is a discrete three-category choice categorical choice and does not depend on the absolute calibration of Likert ratings. Although all 3 responses included explicit reasoning, the reference reasoning was itself model-drafted and physician-verified (produced by the same pipeline as the SFT training targets), so all three candidates shared a similar reasoning style; residual presentation-style differences and the greater completeness of the model outputs may nonetheless have influenced the preference. Therefore, the 3-way best-response finding should be interpreted cautiously and should not be taken as evidence of model superiority over physician decision-making. Third, the field-level subgroup pattern in which GRPO reduced *F*_1_ in ICSI cases is derived entirely from automatic metrics and is independent of any human rating. The convergence across these three independent measurements is the basis for the alignment paradox interpretation, and we discuss the interrater reliability findings that motivate this multisource framing in the Limitations section. The physician evaluation focused on SFT and GRPO because these two models represented the most clinically informative contrast in our experimental setting: SFT served as the supervised reasoning baseline, whereas GRPO achieved the strongest automatic metric performance. A full physician evaluation of all 4 paradigms would further strengthen the clinical interpretation of these findings and should be prioritized in future validation studies.

In addition, in the blinded preference settings of this study, expert reviewers tended to select LLM-generated responses more frequently than the original charted treatment plans. This finding should not be interpreted as evidence that model-generated decisions are clinically superior to real-world physician decision-making. Rather, it likely reflects the value of clear, standardized, and explicitly reasoned output presentation. Original clinical plans in electronic health records are often concise and decision-oriented, whereas model outputs are inherently more verbose and structured. Therefore, comparisons between model outputs and charted treatment plans may be affected by format asymmetry, while the comparison between SFT and GRPO is less affected because both models generated responses in the same format.

### Interpretation of Findings

This reverse pattern is consistent with concerns raised in the broader RLHF and process-supervision literature, where reward overoptimization is known to compress reasoning diversity and amplify confidently stated yet weakly justified outputs. Because our reward function scored only the correctness of final structured fields, GRPO had no explicit incentive to preserve the multistep clinical reasoning that humans evaluated. In complex, multidimensional decision systems, such as infertility treatment, many components of the reasoning chain are interdependent and difficult to evaluate using isolated final-field accuracy alone. Thus, an answer may be statistically correct at the field level while remaining less clinically interpretable, less feasible, or less trustworthy from the perspective of a physician reviewer.

The ICL variant evaluated here used a gated “use-the-guideline-when-uncertain” prompt that relies on the model’s self-assessed epistemic uncertainty. Because contemporary LLMs are known to calibrate this capability poorly, the reported ICL performance reflects this specific prompt configuration rather than a general property of guideline-based prompting. Alternative prompting strategies should be evaluated in future work before drawing broader conclusions about the ICL paradigm in clinical reasoning tasks.

The hallucination analysis also clarifies an apparent tension between the binary hallucination rates and the high Likert-score distribution. Hallucination was operationalized as a sensitive binary safety screen and therefore included low-severity extraneous or unsupported statements, not only clinically dangerous recommendations. Most flagged hallucinations did not alter the final treatment plan, which likely explains why they were not consistently reflected in lower reasoning or feasibility scores. Nevertheless, unsupported content remains clinically undesirable because it may reduce trust, increase cognitive burden, or become consequential in different clinical contexts. Future evaluation rubrics should therefore separate low-impact unsupported statements from safety-critical hallucinations and should explicitly penalize errors that could change diagnosis, ART strategy, COS regimen, or gonadotropin dosing.

The subgroup analyses suggest a hypothesis-generating pattern: GRPO improved *F*_1_ substantially in rare and complex categories such as PGT-M and PGT-SR, but reduced *F*_1_ in ICSI-related scenarios. We attribute this to a data structuring choice in our dataset, male-factor diagnostic parameters such as semen analysis were retained only in unstructured fields, while structured baseline data were predominantly female-factor, which limited the reward signal available for ICSI-relevant decisions. In other words, even when reinforcement learning works as designed, it can amplify rather than correct upstream representational asymmetries in the training data. A complementary interpretation is that ICSI-related decisions may occupy a more ambiguous boundary region between common IVF pathways and more specialized treatment categories. In such settings, reinforcement learning optimized for final-field correctness may favor stable, high-frequency decision patterns rather than preserving sensitivity to individualized edge cases. We did not directly quantify decision-boundary uncertainty, subgroup calibration, or confidence around these overlapping categories; therefore, this interpretation should be regarded as hypothesis-generating. Importantly, this pattern should not be interpreted as evidence of an intrinsic limitation of GRPO or reinforcement learning itself. The observed decline in ICSI-related cases may reflect the interaction between single-center case mix, institution-specific ART decision preferences, male-factor information being retained primarily in unstructured narratives, and the specific static reward configuration used in this study. External validation is required to distinguish these explanations. Future studies should examine whether boundary-focused evaluation, subgroup-sensitive reward scaling, or uncertainty-aware training objectives can reduce this type of performance trade-off.

Together, these findings suggest that accuracy-oriented benchmarks alone may be insufficient for clinical trust alignment. It is not enough for a model to recommend a single correct regimen; it must also demonstrate that the recommendation results from a valid clinical pathway, explicitly ruling out contraindications, preserving relevant patient-specific constraints, and adhering to safety heuristics. Clinical alignment therefore requires evaluation frameworks that jointly assess final-field correctness and the reasoning pathway that produced it.

### Limitations

This study has several limitations. First, this is a single-center retrospective dataset from a single reproductive medical center in Chengdu, China. Patient demographics, treatment-strategy preferences, drug availability, and institutional protocols at this center may not generalize to other regions or to resource-constrained settings. The current input schema captures biomedical and reproductive history, but does not include psychological state, treatment preferences, economic constraints, or detailed longitudinal treatment history. This likely limits model performance on psychologically and socioeconomically complex cases. The observed ICSI subgroup decline under GRPO should be interpreted cautiously as a descriptive and hypothesis-generating finding rather than as evidence of a general property of the GRPO algorithm. This pattern may partly reflect the single-center case mix and institution-specific practice style, because ground-truth diagnoses and treatment plans represent “single-center ground truth”; for clinically plausible cases, different physicians or centers may reasonably annotate different treatment plans based on local protocols, resource availability, and individual treatment philosophy. Beyond these case-mix and annotation-style limitations, the input schema itself was structurally imbalanced: critical male-factor diagnostic parameters such as semen analysis (sperm count, motility, and morphology) were retained only in the unstructured medical-history narrative rather than as structured baseline variables, whereas structured baseline inputs were predominantly female-factor. Because semen analysis is a primary diagnostic criterion for selecting ICSI and related procedures such as TESA+ICSI and IVF with donor sperm, this asymmetry left the model dependent on unstructured-text extraction for male-factor reasoning, and is one plausible contributor to the GRPO performance decline observed in the ICSI subgroup. In addition, because GRPO was trained using a static reward configuration, the observed algorithmic-clinical decoupling may be partly dependent on this specific reward design. Formal reward-weight ablation and external multicenter validation are needed to determine whether the same pattern persists under alternative reward configurations and institutional practice settings. Second, ChatGPT-4o served several compounding roles in this study. It was used to translate unstructured Chinese clinical narratives into English, to generate the CoT reasoning used for SFT, and to serve as the auxiliary semantic judge for the initial-diagnosis field. Therefore, part of the alignment benchmark was model-derived rather than fully independent human-authored clinical logic. This design may introduce teacher-model stylistic bias, residual translation error in reproductive-endocrinology terminology, and evaluation bias toward phrasing patterns preferred by the semantic judge. To mitigate these risks, the CoT prompts were curated by reproductive medicine specialists, the generated reasoning examples were reviewed during dataset construction, and the translation prompts instructed the model to retain medically important information, use professional or standard medical terminology, and translate accurately and concisely; the medical-history prompt additionally specified that the translation should be produced without speculation. In addition, we conducted a bounded bilingual translation audit in which a reproductive medicine specialist reviewed a random subset of 50 translated narratives against the original Chinese records; the audit identified 1 terminology error, 0 numerical errors, 0 omissions or additions, and 0 clinically consequential errors. Although this audit contextualizes the magnitude of translation risk, it does not eliminate the possibility that model-derived reasoning or model-based semantic judging influenced the observed differences between alignment strategies. Future multi-center validation should use independently human-authored reasoning labels, bilingual expert-verified source narratives, and semantic-judge validation against blinded expert adjudication. Third, the blinded independent expert review relied on two reproductive medicine specialists. The 2 reviewers agreed within 1 Likert point on 93.7% of pooled ratings, with PABAK of 0.26 and Gwet AC1 of 0.32 pooled (fair to moderate on the Landis and Koch scale). Raw Cohen κ was near zero because rating distributions were strongly concentrated on the upper end of the Likert scale, consistent with the well-described kappa paradox. We mitigate this 3 ways. First, we report Holm-adjusted *P* values (Wilcoxon signed-rank, *P*=.07 for reasoning capability and *P*=.06 for treatment feasibility) and paired effect sizes (Cohen *d*=+0.22 and+0.25, small). Second, the directional pattern is cross-validated by the discrete 3-way best-response preference (SFT 51.2% vs GRPO 26.2% vs charted plan 22.6%), which does not depend on absolute Likert agreement. Third, the field-level subgroup pattern is independently supported by automatic metrics. Expanding the reviewers and including geographically diverse subspecialty experts with a more discriminating evaluation instrument remains an explicit priority for follow-on multicenter validation. Fourth, Likert-scale ratings are ordinal; we additionally report medians and IQRs and note that parametric effect sizes (Cohen *d*) are presented under the assumption of continuous Likert data, which we treat as a study limitation rather than as a claim of interval-level measurement. Fifth, the absolute hallucination rates observed in this study (18.5% for SFT and 15% for GRPO) pose substantial safety risks in a high-stakes domain such as reproductive endocrinology, where a single clinically incorrect or unsupported statement can translate into an inappropriate stimulation protocol, an inappropriate dosage, or an inappropriate procedure selection. Although the postalignment GRPO model showed a relatively lower hallucination rate, the absolute level remains far above any threshold compatible with autonomous or even assistant-mode clinical deployment, and the relative reduction should not be interpreted as evidence of clinical safety. We position all 4 models studied here as research artifacts for evaluating alignment strategy and not as clinical tools. Any future clinical translation of this work should be preceded by safety-critical error grading, calibration analysis, controlled clinician-in-the-loop deployment trials, and explicit fallback or refusal mechanisms when the model’s confidence is insufficient. Sixth, the comparison between the model-generated responses and the original physician-charted plans may be affected by format asymmetry. In the reference response, the diagnosis and treatment plan were the genuine physician-charted ground truth, but the accompanying reasoning was not independently human-authored: it was produced by the same ChatGPT-4o–based, specialist-curated pipeline used to create the SFT training targets and was physician-verified. All 3 candidate responses therefore shared a similar explicit-reasoning style, so residual presentation-style differences, together with the greater completeness of the model outputs relative to the concise charted plans, may have inflated reviewer preference for the model responses. Any preference for model outputs over charted plans should thus be interpreted as reflecting presentation quality and reasoning completeness rather than clinical superiority over physician decision-making; notably, the SFT baseline was preferred even more often than the reference response itself (51.2% vs 22.6%). This also means the alignment benchmark is in part model-derived. The SFT-versus-GRPO comparison is less affected by this limitation because both models generated outputs in the same format. Finally, the ICL evaluation relied on a single gated prompt configuration that asked the model to consult the guideline only when uncertain about its answer. Because contemporary LLMs are known to calibrate epistemic uncertainty poorly, the underperformance of ICL relative to SFT in our study should be attributed to this specific prompt implementation rather than to the ICL paradigm in general. Systematic comparison against unconditional guideline conditioning, retrieval-augmented prompting, and chain-of-verification approaches remains a priority for follow-on work.

### Future Directions

Translating these findings into clinical practice will require a phased validation pathway. The immediate next step is external multi-center prospective validation across centers with different case-mix and protocol preferences. A second step is integration with electronic health record systems so that model reasoning chains can be inspected at the point of care, rather than after the fact. A third step is testing dynamic decision-making in longitudinal scenarios such as follicle monitoring, where treatment decisions are revised as new information arrives. Additionally, incorporate multimodal patient-profile inputs, including psychological state, treatment-preference elicitation, socioeconomic context, and longitudinal treatment history, to build more comprehensive ART decision-support systems. Beyond the decision-support role studied here, future iterations should also incorporate referral markers (eg, automatically recommending genetic counseling for patients with a history of recurrent miscarriage) so that the system reflects the multidisciplinary structure of reproductive medicine care. Methodologically, future work should pair process-level and outcome-level rewards within a single training objective, conduct ablation studies on reward weighting, and extend evaluation dimensions of “treatment feasibility” into operational subdimensions such as drug availability, monitoring burden, and the impact on patients’ work and personal life. Future reward design should also incorporate structured ART guidelines and clinical constraints, so that inconsistent reasoning steps, unsupported causal jumps, contraindication violations, and deviations from established treatment pathways can be explicitly penalized rather than ignored by final-answer scoring. Future evaluation frameworks should also move beyond overall accuracy to include fine-grained clinical fairness and subgroup robustness. Clinically important margin cases, such as stimulation risk profiles, embryo-yield expectations, dosing safety margins, and adherence to OHSS-prevention heuristics, may provide a more operationally meaningful assessment of whether model recommendations remain safe and useful near decision boundaries. Finally, future studies should examine whether the Alignment Paradox persists across model scales. Larger medical LLM backbones may encode richer clinical context and may differ in their sensitivity to reward-induced distributional shifts, but this remains an empirical question rather than an assumption.

### Conclusions

In this single-center retrospective study, automatic metrics were compared across four alignment paradigms, whereas clinician-perceived reasoning quality was evaluated in the SFT versus GRPO contrast. GRPO achieved the strongest automatic field-level performance, while SFT showed directionally higher expert ratings for reasoning capability and treatment feasibility, and the absolute hallucination rate of both models remained too high for clinical deployment. Outcome-based metrics alone are therefore insufficient proxies for clinical utility in ART AI systems, and external validation paired with process-level evaluation is required before any deployment claim can be supported. Because the clinical review was based on two reproductive medicine specialists with marginal inter-rater agreement, conclusions about clinical alignment should be interpreted as exploratory and require confirmation in larger multi-center expert panels.

## Supplementary material

10.2196/97221 Multimedia Appendix 1 In-context learning guideline blocks, reproducibility details, direct preference optimization epoch sensitivity analysis, and full doctor-in-the-loop evaluation statistics.

10.2196/97221 Multimedia Appendix 2 Doctor-in-the-loop evaluation. (A) Expert-rated performance across three dimensions (5-point Likert scale and mean of 2 raters). Supervised fine-tuning (SFT) shows consistently higher reasoning and feasibility scores (Holm-adjusted *P*=.07 and .06, respectively); group relative policy optimization (GRPO) has a descriptively lower hallucination percentage. (B) Blinded best-response selection across SFT, GRPO, and physician-charted plans (GT). SFT won more often than both GRPO (*P*<.001) and GT (*P*<.001); the difference between GRPO and GT was not statistically significant (*P*=.48).

10.2196/97221 Multimedia Appendix 3 Subgroup performance across 4 alignment strategies and 3 major assisted reproductive technology categories: in vitro fertilization (IVF), intracytoplasmic sperm injection (ICSI), preimplantation genetic testing (PGT), in the held-out test set (single-center retrospective dataset). *F*_1_-scores are displayed on a 0-1 scale. Group relative policy optimization improved *F*_1_ in IVF and PGT but reduced *F*_1_ in ICSI, where male-factor diagnostic parameters were captured primarily in unstructured fields.

## Abbreviations

ART: assisted reproductive technology
CC: clomiphene citrate
COS: controlled ovarian stimulation
CoT: chain-of-thought
DPO: direct preference optimization
EHR: electronic health record
GnRH: gonadotropin-releasing hormone
GRPO: group relative policy optimization
HIPAA: Health Insurance Portability and Accountability Act
ICL: in-context learning
ICSI: intracytoplasmic sperm injection
IVF: in vitro fertilization
LLM: large language model
LoRA: low-rank adaptation
MAE: mean absolute error
OHSS: ovarian hyperstimulation syndrome
PABAK: prevalence-adjusted bias-adjusted kappa
PGT: preimplantation genetic testing
PGT-A: preimplantation genetic testing for aneuploidy
PGT-M: preimplantation genetic testing for monogenic disorders
PGT-SR: preimplantation genetic testing for structural rearrangements
PPO: proximal policy optimization
PPOS: progestin-primed ovarian stimulation
RLHF: reinforcement learning from human feedback
RQ: research question
SFT: supervised fine-tuning
SOTA: state-of-the-art
TESA: testicular sperm aspiration
WHO: World Health Organization
