# Supplementary material for: The Alignment Paradox of Medical Large Language Models in Infertility Care: Decoupling Algorithmic Improvement From Clinical Decision-Making Quality
Source: J Med Internet Res. 2026 Sep 21;28:e97221. doi: 10.2196/97221 (PMC13592391; doi:10.2196/97221)
Supplement: Supplementary file 1 [file jmir-v28-e97221-s001.docx]

# Appendix S1. ICL Guideline Blocks

Two natural-language clinical decision blocks embedded into the in-context learning (ICL) prompt during inference. Guidelines were derived from physician-validated decision flowcharts used at the Reproductive Medical Center, West China Second University Hospital, Sichuan University.

## S1.1 Clinical Decision Guidelines for Assisted Reproduction Strategy

Here is the list of clinical decision pathways. Please make sure to choose the right one:

### 1. IVF

IVF is the primary recommendation for the following conditions:

- The patient has confirmed pelvic or tubal factors, such as tubal obstruction, a history of tubal surgery, or severe endometriosis.
- The patient has had bilateral fallopian tube removal or ligation.
- The patient has a history of secondary infertility (has been pregnant before) and is either over 35 years old or has been trying to conceive for over 3 years.
- The patient has unexplained infertility and a Short Protocol IVF is being considered.

### 2. ICSI

ICSI is the primary recommendation for the following conditions:

- The male partner has been diagnosed with severe oligoasthenoteratozoospermia (severe OATs; low sperm count, motility, and morphology).
- The patient had a previous IVF cycle with abnormal or failed fertilization.

As a general principle, if a patient’s previous cycle was ICSI, the subsequent cycle should also be ICSI, unless the male partner’s semen analysis has significantly improved to normal levels.

### 3. PGT

PGT-A is indicated for patients with:

- Advanced maternal age.
- A history of recurrent miscarriage.
- Known chromosomal number abnormalities (aneuploidy) or sex chromosome abnormalities.
- A history of recurrent implantation failure (as an option based on patient preference).

PGT-M is indicated for patients with:

- Known monogenic (single-gene) diseases.
- Cases where both partners are carriers of the same pathogenic gene mutation.

PGT-SR is indicated for patients with:

- Known structural chromosome abnormalities or rearrangements (e.g., translocations).

### 4. Specialized & Combined Procedures

TESA+ICSI (Testicular Sperm Aspiration + ICSI): This is specifically for males diagnosed with azoospermia (no sperm in ejaculate) where viable sperm are successfully retrieved via testicular puncture or microdissection.

IVF with Donor Sperm / ICSI with Donor Sperm:

This is indicated for males with azoospermia where no sperm can be retrieved via surgical methods, or when the patient declines surgery. The choice between IVF or ICSI with donor sperm would then depend on the female partner’s condition and oocyte quality.

Short Protocol IVF:

This specific IVF protocol is often preferred for:

- Patients with unexplained infertility or moderate male factor infertility.
- Patients with primary infertility who are over 35 years old or have been trying for over 3 years.
- Patients who have had ≥3 failed AIH cycles.

## S1.2 Clinical Decision Guidelines for COS Regimen

Here is the list of COS regimens. Please make sure choosing the right one. The GnRH Antagonist Protocol is not always appropriate.

**1. GnRH Antagonist Flexible Protocol**

A protocol using GnRH antagonists initiated based on follicular development, offering cycle flexibility and reducing the risk of ovarian hyperstimulation syndrome (OHSS).

**2. Luteal Short-Acting Long Protocol**

Involves administration of short-acting GnRH agonists starting in the luteal phase to achieve pituitary downregulation prior to gonadotropin stimulation.

**3. GnRH Antagonist Fixed Protocol**

A protocol where GnRH antagonists are started on a fixed day (typically day 5 or 6 of stimulation) to prevent premature LH surge during controlled ovarian stimulation.

**4. Follicular Phase Long-Acting Protocol**

Involves administration of long-acting GnRH agonists in the follicular phase for sustained pituitary suppression before gonadotropin administration.

**5. Progestin-Primed Ovarian Stimulation Protocol (PPOS)**

Uses oral progestins instead of GnRH analogs to suppress the LH surge during ovarian stimulation, often employed in freeze-all cycles.

**6. Clomiphene Citrate Plus Gonadotropins**

Combines oral clomiphene citrate with injectable gonadotropins to stimulate follicular development with lower gonadotropin requirements.

**7. Mild Stimulation Protocol / Direct Gn Protocol**

Aims to retrieve fewer, high-quality oocytes using lower doses of gonadotropins, with or without oral agents like clomiphene or letrozole.

**8. Luteal Phase Stimulation Protocol**

Ovarian stimulation initiated during the luteal phase of the menstrual cycle, often used in dual stimulation (“DuoStim”) strategies for poor responders.

**9. Clomiphene or Letrozole Combined with Gonadotropins**

Combines oral ovulation induction agents (clomiphene or letrozole) with gonadotropins to enhance follicular recruitment in a cost-effective manner.

**10. Conventional Ultra-Long Protocol**

Involves extended administration of long-acting GnRH agonists (≥2–3 months) before ovarian stimulation, typically for endometriosis or adenomyosis patients.

**11. Modified Ultra-Long Protocol**

A variant of the ultra-long protocol with adjusted timing or dosage of GnRH agonist to optimize response while minimizing side effects.

**12. Short Protocol (GnRH-a Short Protocol)**

Uses a short-acting GnRH agonist starting in the early follicular phase alongside gonadotropins, leveraging a transient flare-up effect to enhance follicle recruitment.

# Appendix S2. Reproducibility Details

This appendix documents the exact software framework, model versions, training hyperparameters, prompt templates, and decoding settings required to reproduce the four alignment paradigms (SFT, DPO, GRPO, ICL) and the auxiliary evaluators reported in the main manuscript.

## S2.1 Software framework and backbone

- Training framework for SFT and DPO: LLaMA-Factory (https://github.com/hiyouga/LLaMA-Factory).
- Training framework for GRPO: verl (https://github.com/volcengine/verl), PPO trainer with algorithm.adv_estimator = grpo.
- Inference framework for evaluation rollouts inside GRPO: vLLM (bfloat16, tensor-model-parallel size 2).
- CoT generation, narrative translation, and auxiliary semantic judge: OpenAI ChatGPT (model identifier: gpt-4o); details in A2.6, A2.7, A2.8.
- Backbone model: aaditya/Llama3-OpenBioLLM-8B (HuggingFace identifier).
- Random seeds and decoding parameters: framework default values where not otherwise specified.

## S2.2 SFT training configuration

SFT was performed using the LLaMA-Factory framework (https://github.com/hiyouga/LLaMA-Factory) with mostly default settings. All hyperparameters not listed below use the LLaMA-Factory default value at the time of training.

- Backbone: aaditya/Llama3-OpenBioLLM-8B (HuggingFace identifier).
- Adapter: LoRA, rank = 8, alpha = 16, dropout = 0.0 (LLaMA-Factory defaults).
- LoRA target modules: "all" (LLaMA-Factory default; expands to q_proj, k_proj, v_proj, o_proj, gate_proj, up_proj, down_proj for Llama-family models).
- Optimizer: adamw_torch (LLaMA-Factory default; AdamW with β₁ = 0.9, β₂ = 0.999, ε = 1e-8).
- Learning-rate scheduler: cosine (LLaMA-Factory default).
- Warmup ratio: 0.0 (LLaMA-Factory default).
- Mixed precision: bf16.
- Learning rate: 3e-5.
- Per-device train batch size: 4.
- Epochs: 10.
- Checkpoint selection: validation-set loss plateau at epoch 10.
- Hardware: 1 × NVIDIA A100 80GB.

## S2.3 DPO training configuration

DPO was also performed using the LLaMA-Factory framework with the same LoRA adapter configuration as SFT (rank = 8, alpha = 16, dropout = 0.0, target modules = all). Hyperparameters not listed below use the LLaMA-Factory default value.

- Reference policy: SFT checkpoint described in A2.2.
- Optimizer: adamw_torch (LLaMA-Factory default).
- Learning-rate scheduler: cosine (LLaMA-Factory default).
- Mixed precision: bf16.
- Learning rate: 3e-7.
- Per-device train batch size: 8.
- DPO β: 0.3.
- Epochs: 1 (reported configuration).
- Sensitivity analysis: additionally trained at 2 epochs and at 10 epochs with early stopping on validation loss; results in Multimedia Appendix 3.
- Hardware: 1 × NVIDIA A100 80GB.

## S2.4 GRPO training configuration

GRPO was trained with the verl framework. The exact training command, with environment variables resolved, was:

```
python3 -m verl.trainer.main_ppo \
    algorithm.adv_estimator=grpo \
    data.train_files=/workspace/ubuntu/train.parquet \
    data.val_files=/workspace/ubuntu/val.parquet \
    data.train_batch_size= 16 \
    data.val_batch_size= 16 \
    data.max_prompt_length=512 \
    data.max_response_length=1024 \
    data.filter_overlong_prompts=True \
    data.truncation='error' \
    data.shuffle=False \
    actor_rollout_ref.model.path=$MODEL_PATH \
    actor_rollout_ref.model.use_shm=False \
    actor_rollout_ref.rollout.dtype=bfloat16 \
    actor_rollout_ref.model.enable_gradient_checkpointing=True \
    actor_rollout_ref.model.lora_rank=64 \
    actor_rollout_ref.model.lora_alpha=32 \
    actor_rollout_ref.model.target_modules=all-linear \
    actor_rollout_ref.actor.optim.lr=3e-5 \
    actor_rollout_ref.model.use_remove_padding=True \
    actor_rollout_ref.actor.ppo_mini_batch_size=${mini_batch_size} \
    actor_rollout_ref.actor.ppo_micro_batch_size=${mini_batch_size} \
    actor_rollout_ref.actor.use_kl_loss=True \
    actor_rollout_ref.actor.kl_loss_coef=0.001 \
    actor_rollout_ref.actor.kl_loss_type=low_var_kl \
    actor_rollout_ref.actor.fsdp_config.fsdp_size=-1 \
    actor_rollout_ref.actor.fsdp_config.param_offload=True \
    actor_rollout_ref.actor.fsdp_config.optimizer_offload=True \
    actor_rollout_ref.rollout.log_prob_micro_batch_size=${mini_batch_size} \
    actor_rollout_ref.rollout.tensor_model_parallel_size=2 \
    actor_rollout_ref.rollout.name=vllm \
    actor_rollout_ref.rollout.gpu_memory_utilization=0.8 \
    actor_rollout_ref.rollout.n=5 \
    actor_rollout_ref.rollout.max_num_seqs=256 \
    actor_rollout_ref.rollout.max_model_len=2048 \
    actor_rollout_ref.rollout.max_num_batched_tokens=2048 \
    actor_rollout_ref.rollout.enable_chunked_prefill=True \
    actor_rollout_ref.rollout.load_format=safetensors \
    actor_rollout_ref.rollout.layered_summon=True \
    actor_rollout_ref.ref.log_prob_micro_batch_size=${mini_batch_size} \
    actor_rollout_ref.ref.fsdp_config.param_offload=True \
    actor_rollout_ref.actor.ulysses_sequence_parallel_size=2 \
    actor_rollout_ref.actor.entropy_coeff=0.001 \
    algorithm.kl_ctrl.kl_coef=0.001 \
    algorithm.use_kl_in_reward=False \
    trainer.critic_warmup=0 \
    trainer.logger=['console','wandb'] \
    trainer.n_gpus_per_node=4 \
    trainer.nnodes=1 \
    trainer.save_freq=50 \
    trainer.test_freq=10 \
    trainer.total_epochs=5 \
    trainer.resume_mode=disable \
    custom_reward_function.path=/workspace/ubuntu/medical_reward.py \
    custom_reward_function.name=compute_score
```

Reward function summary. The compute_score function combines four per-field accuracy rewards as a weighted sum: R = 0.2 × R_InfType + 0.3 × R_ART + 0.3 × R_COS + 0.2 × R_Gn. The Gn-dose reward is graded: 1.0 if |ΔGn| ≤ 25 IU; 0.5 if 25 IU < |ΔGn| ≤ 50 IU; 0.0 otherwise. The PPO clipping ratio ε was kept at the verl default value of 0.2.

## S2.5 ICL guideline-based prompt template

The two guideline blocks shown in Multimedia Appendix 1 were inserted into the SFT inference prompt under the following gating instruction, with max output 2048 token:

SYSTEM:

You are a reproductive medicine specialist. Below are two clinical

decision guideline blocks.

[Guideline A: ART Strategy]

{A1.1}

[Guideline B: COS Regimen]

{A1.2}

USER:

DO NOT USE THE GUIDELINE UNLESS YOU ARE NOT SURE ABOUT YOUR ANSWER.

Patient information:

{structured_baseline}

{unstructured_history}

{unstructured_ultrasound}

Please output, in the following order:

1) Infertility type (Primary / Secondary / Other)

2) Initial differential diagnosis

3) ART strategy

4) COS regimen

5) Gonadotropin starting dose (IU)

Provide a reasoning chain before each decision.

## S2.6 CoT generation prompt template (SFT dataset curation)

Chain-of-thought (CoT) reasoning used for SFT training was generated by ChatGPT (model identifier: gpt-4o, the same model used for translation and for the auxiliary semantic judge described in A2.7 and A2.8) using a six-shot selective few-shot prompting strategy. The same CoT generation methodology, including the three prompting strategies that were compared (zero-shot, random few-shot, and selective few-shot) and the three-dimensional 5-point Likert evaluation rubric used to validate them, has been previously published by our group [Liu et al., 2026, J. Med. Internet Res. 28:e85206, reference 27 in the main manuscript]. In that prior validation study, the selective few-shot prompt significantly outperformed both the zero-shot and the random few-shot prompts across the PGT, ICSI, and IVF subgroups on all three rubric dimensions (Logical Coherence and Consistency [LCC], Plausibility of Clinical Argumentation and Rationale [PCAR], and Utilization of Critical and Key Information [UCKI]), with Holm- and FDR-adjusted P values consistently below .05 and most below .001. The selective few-shot prompt template was therefore adopted for the present study.

The exact system prompt used in the present study was:

```
You are a highly experienced reproductive medicine specialist.
Given the patient information and the actual final treatment plan
decided by a human expert, please provide a clear and concise
step-by-step reasoning process (Chain-of-Thought) that logically
explains how one could arrive at the given treatment plan and
diagnosis based on the patient's condition. Your reasoning must
reflect expert-level clinical decision-making and strictly follow
the given structure. Avoid unnecessary elaboration, and keep the
reasoning focused and succinct.

The reasoning must be structured into four parts:
  1. Diagnostic Basis
  2. Assisted Reproductive Strategy
  3. Ovarian Stimulation Protocol
  4. Initial Gonadotropin Dose

Please begin your response directly with "Analysis:". Reason
authentically as a doctor rather than explaining the output, and
keep the chain of thought concise and to the point.
```

Six in-context examples were appended to the system prompt. The six exemplar cases were selected by a reproductive medicine specialist to span the major decision pathways of the dataset: (1) primary infertility with PCOS leading to Short Protocol IVF, (2) secondary infertility with diminished ovarian reserve leading to IVF, (3) primary infertility with male-factor azoospermia leading to IVF with Donor Sperm, (4) primary infertility with severe teratozoospermia leading to ICSI, (5) primary infertility with obstructive azoospermia leading to TESA+ICSI, and (6) recurrent miscarriage with prior embryonic aneuploidy leading to PGT-A. The full six-example template, including all input fields, the reasoning chains, and the ground-truth treatment plans, is reproduced verbatim in Appendix A2 of Liu et al. (2026). A single representative example is reproduced below for inline reference:

```
--- Example 1 (PCOS, primary infertility, Short Protocol IVF) ---

【Patient Information】
- Age: 32 years
- Menstrual Cycle: 20-30 days
- Weight: 48.0 kg
- BMI: 17.42
- AMH: 7.56 ng/mL
- FSH: 6.3
- Infertility Duration: 6.0 years
- Ultrasound Findings: Homogeneous endometrial echo and
  suspicious polycystic changes in both ovaries
- Medical History Summary: [verbatim narrative from the source
  EHR, abbreviated here for length]

【Final Treatment Plan Provided】
- Infertility Type: Primary infertility
- Initial Diagnosis: Polycystic ovary syndrome
- COS Protocol: GnRH Antagonist Flexible Protocol
- Initial Gn Dose: 125 IU/day
- Treatment Method: Short Protocol IVF

【Reasoning Process to Reach the Provided Plan】
Analysis:
1. Diagnostic Basis:
   The couple has engaged in regular unprotected sexual intercourse
   for 6 years without conception and has no history of prior
   pregnancy, consistent with primary infertility. The patient has
   a previous diagnosis of PCOS, with current menstrual
   irregularities and ultrasound findings of polycystic ovarian
   morphology, supporting this diagnosis. HSG shows bilateral
   tubal patency. According to the WHO 6th edition criteria, the
   male partner's semen analysis is normal.
2. Assisted Reproductive Strategy:
   Given the history of infertility, patent fallopian tubes, and
   normal semen analysis, intrauterine insemination is a feasible
   first-line option. However, considering primary infertility,
   long duration of infertility, and previous AIH failure,
   Short Protocol IVF is recommended; rescue ICSI may be used if
   fertilization fails.
3. Ovarian Stimulation Protocol:
   The female partner is <35 years old with PCOS and elevated AMH,
   indicating high ovarian response potential. A GnRH antagonist
   protocol is preferred to reduce OHSS risk.
4. Initial Gonadotropin Dose:
   For patients with high ovarian response potential, 100-150 IU
   is recommended. Given low body weight (48 kg) and underweight
   BMI (<18), a lower starting dose of 125 IU is selected to
   minimize OHSS risk.
```

Quality control. CoT outputs that failed to follow the four-part structure or that contradicted the ground-truth treatment plan were filtered automatically. The validation rubric, including the operational definitions of LCC, PCAR, and UCKI and the 5-point Likert anchors, is reproduced in Appendix A3 of Liu et al. (2026). Representative annotation examples are reported in the same prior publication.

## S2.7 Chinese-to-English translation prompt templates

Three distinct prompts were used to translate the three unstructured Chinese fields (medical history, female initial diagnosis, and gynecological ultrasound). All three were run with ChatGPT (model identifier: gpt-4o) at temperature 0.4. A bounded bilingual translation audit was performed within the present study: a reproductive medicine specialist reviewed a random subset of 50 translated narratives against the original Chinese records and identified 1 terminology error, 0 numerical errors, 0 omissions or additions, and 0 clinically consequential errors. The implications of residual translation risk are discussed in the Limitations section of the main manuscript.

**S2.7.1 Medical-history translation prompt.**

```
You are a physician assistant who specializes in assisted
reproduction. Please follow the steps below to process the Chinese
medical history below:
1. **You must Remove all statements that patient asks (or doctor
   recommendation) for IVF or ICSI, such as "要求IVF助孕",
   "要求ICSI助孕", "PGT-M助孕", "TESA助孕", "PGT-A助孕",
   "PGT-SR助孕", "PGS助孕", "FET助孕", "PGD助孕", etc. or you will
   be penalized.**
2. Retain all medically important information that may affect
   diagnosis or treatment.
3. Translate the retained medical history into professional medical
   English, accurately and concisely, without speculation.
4. The final output should be a description of the medical history
   in English.

The medical history is as follows:
{history_text}
```

Rationale for step 1: the original Chinese narratives often record the patient’s or referring physician’s stated treatment preference (for example, “the patient requests IVF”). Including such statements in the SFT input would leak treatment information into a field that should describe disease state only. The instruction therefore strips these phrases before translation.

**S2.7.2 Female initial-diagnosis translation prompt.**

```
You are a physician assistant who specializes in assisted
reproduction. Please follow the steps below to process the Chinese
female diagnosis below:
**1. Translate the diagnosis into professional medical English,
     accurately and concisely. Remove all the redundancy. Or you
     will be penalized.**
2. Maintain all medically important information.
3. Use standard medical terminology.
4. The final output should be a clear and concise English
   diagnosis.

The diagnosis is as follows:
{diagnosis_text}
```

**S2.7.3 Gynecological ultrasound translation prompt.**

```
You are a physician assistant who specializes in assisted
reproduction. Please follow the steps below to process the Chinese
female ultrasound description below:
**1. Translate the ultrasound result into professional medical
     English, accurately and concisely. Remove all the redundancy.
     Or you will be penalized.**
2. Maintain all medically important information.
3. Use standard medical terminology.
4. The final output should be a clear and concise English
   diagnosis.
**5. If the source says "无特殊" or "-", just say "No special
     results".**

The diagnosis is as follows:
{ultrasound_text}
```

## S2.8 Auxiliary LLM semantic judge prompt template

The initial differential diagnosis field exhibits high paraphrasing variability and exact-match evaluation is therefore unreliable. We used ChatGPT (model identifier: gpt-4o) as an auxiliary semantic judge that produces two binary indicators per case: a "partial" match (at least one ground-truth diagnosis appears in the predicted list) and an "exact" match (all ground-truth diagnoses are present in the predicted list, with paraphrased or more specific forms counted as matches). These two indicators correspond to the "Partial" and "Exact" columns reported under "Initial diagnosis" in Table 1 of the main manuscript. The exact judge prompt is reproduced below.

```
You are a medical evaluator and experienced in assisted
reproduction. Your task is to determine whether the predicted
diagnoses match the ground truth diagnoses from two criteria:

Ground Truth Diagnoses:
{gt_diag}

Predicted Diagnoses:
{pred_diag}

Please follow these instructions:

1. **at_least_one_match**: Is there at least one diagnosis in the
   predicted list that is also in the ground truth list?
2. **all_match**: Does the predicted list contain all diagnoses
   from the ground truth list? Some diagnoses may be paraphrased
   but remain the same meaning, and you need to consider them as
   correct. For example, if the predicted one contains
   "Chromosomal abnormality in the female partner." and the ground
   truth is "Chromosomal abnormality", this should be considered
   as true. Count it as match if the predicted diagnosis
   **includes** the ground truth either explicitly or in a **more
   specific form**.

Respond in the following JSON format:
{
  "at_least_one_match": 1 or 0,
  "all_match": 1 or 0
}
```

Decoding parameters: temperature = 0, max_tokens = 300. Responses that did not contain a parseable JSON block were retried up to three times with exponential backoff; unparseable responses after retry were assigned the sentinel value −1 and excluded from the averaged "Partial" and "Exact" rates. Limitations of this judge, including potential bias toward phrasing styles that resemble the judge model’s preferred linguistic patterns, are discussed in the Limitations subsection of the main manuscript.

# Appendix S3. DPO Epoch Sensitivity Analysis

To rule out undertraining as a confounder of the relative DPO underperformance, DPO was trained under three configurations on the same preference dataset with identical hyperparameters (β = 0.3, learning rate = 3e-7, batch size = 8): the reported 1-epoch configuration, a 2-epoch configuration, and a 10-epoch configuration with early stopping on validation loss. Field-level accuracy on the held-out 821-case test set degraded sharply rather than improved as training proceeded, consistent with the well-documented DPO collapse phenomenon. The 1-epoch checkpoint was retained as the reported DPO configuration.

| **Field** | **DPO 1 epoch (reported)** | **DPO 2 epochs** | **DPO 10 epochs + early stop** |
| --- | --- | --- | --- |
| Infertility-type accuracy (%) | 92.2 | 72.0 | 53.6 |
| COS regimen accuracy (%) | 60.9 | 38.0 | 18.0 |
| ART strategy accuracy (%) | 75.03 | 12.0 | 9.4 |
| Gn starting-dose MAE (IU) | 45.12 | 55.50 | 58.45 |

*Note. All values computed on the 821-case held-out test set. Lower MAE is better; higher accuracy is better. Across all four metrics, additional DPO training degraded performance, indicating that the relative underperformance of DPO is not attributable to undertraining.*

# Appendix S4. Doctor-in-the-loop Evaluation: Full Statistics

This appendix reports the full descriptive statistics, inter-rater reliability metrics, and pairwise SFT-versus-GRPO comparisons for the blinded independent expert review by two reproductive medicine specialists on 100 randomly selected cases from the held-out test set.

## S4.1 Descriptive statistics

Per-dimension descriptive statistics computed on the per-case mean of the two raters.

| **Dimension** | **SFT median (IQR)** | **SFT mean (SD)** | **GRPO median (IQR)** | **GRPO mean (SD)** |
| --- | --- | --- | --- | --- |
| Diagnostic Accuracy | 4.00 (4.00–4.50) | 4.04 (0.42) | 4.00 (3.50–4.50) | 3.99 (0.45) |
| Reasoning Capability | 4.00 (4.00–4.50) | 4.17 (0.42) | 4.00 (4.00–4.50) | 4.08 (0.42) |
| Treatment Feasibility | 4.50 (4.00–4.50) | 4.21 (0.39) | 4.00 (4.00–4.50) | 4.09 (0.42) |

## S4.2 Inter-rater reliability

Because the marginal Likert distribution was strongly concentrated on the upper end of the scale (62.3% of pooled ratings were 4 and 23.8% were 5), raw Cohen’s kappa is uninformative in this regime (the well-described kappa paradox). We therefore report five complementary metrics per dimension and pooled across the three Likert dimensions.

| **Dimension** | **Pₒ (exact)** | **Pₒ (±1 point)** | **Weighted κ** | **PABAK** | **Gwet’s AC1** |
| --- | --- | --- | --- | --- | --- |
| Diagnostic Accuracy | 0.44 | 0.93 | 0.04 | 0.30 | 0.35 |
| Reasoning Capability | 0.42 | 0.94 | 0.03 | 0.28 | 0.33 |
| Treatment Feasibility | 0.37 | 0.95 | 0.06 | 0.21 | 0.28 |
| Pooled (all three) | 0.41 | 0.94 | 0.05 | 0.26 | 0.32 |

*Notes. Pₒ = raw observed agreement; Pₒ (±1 point) = agreement within one Likert point; Weighted κ = quadratic-weighted Cohen’s kappa; PABAK = prevalence-adjusted bias-adjusted kappa; AC1 = Gwet’s AC1 coefficient. PABAK and AC1 are robust to skewed marginal distributions and fall in the fair-to-moderate range on the Landis and Koch scale.*

## S4.3 Pairwise SFT-vs-GRPO comparisons

Wilcoxon signed-rank tests on the per-case mean of the two raters, with Holm-Bonferroni correction across the three Likert dimensions. Effect sizes are reported as rank-biserial correlation and paired Cohen’s d (with the limitations of treating ordinal Likert data as continuous explicitly noted in the Limitations section of the main manuscript).

| **Dimension** | **W+** | **W−** | **Raw P** | **Holm-adj. P** | **r / d** |
| --- | --- | --- | --- | --- | --- |
| Diagnostic Accuracy | 472 | 308 | .208 | .208 | 0.21 / +0.13 |
| Reasoning Capability | 966 | 520 | .033 | .070 | 0.30 / +0.22 |
| Treatment Feasibility | 1173 | 597 | .015 | .055 | 0.33 / +0.25 |

*Notes. W+ and W− are the sums of positive and negative ranks. r = rank-biserial correlation (non-parametric effect size); d = paired Cohen’s d (parametric effect size, presented under the assumption of continuous Likert data).*

## S4.4 Three-way best-response selection (tie-handling rule)

In the three-way best-response question, reviewers were instructed to identify the single best response among the SFT output, the GRPO output, and the original physician-charted plan. In a small number of cases, reviewers indicated a tie between two responses; for these tied votes the preference was split equally between the tied options (0.5/0.33 to each). Per-rater fractional preferences were then averaged across the two reviewers. The three-way percentages and the pairwise proportions reported in the main manuscript Results section follow this aggregation rule. Tied-vote pseudo-counts (such as the previously reported "102.3 of 154.7" pairwise denominator) have been removed from the main manuscript; the three-way percentages (SFT 51.2%, GRPO 26.2%, charted plan 22.6%) and the pairwise proportions with Holm-adjusted P values are retained.
